# Dynactin-1 mediates rescue of impaired axonal transport due to reduced mitochondrial bioenergetics in amyotrophic lateral sclerosis motor neurons

**DOI:** 10.1093/braincomms/fcae350

**Published:** 2024-10-05

**Authors:** Ruxandra Dafinca, Carlota Tosat-Bitrian, Emily Carroll, Björn F Vahsen, Javier Gilbert-Jaramillo, Jakub Scaber, Emily Feneberg, Errin Johnson, Kevin Talbot

**Affiliations:** Nuffield Department of Clinical Neurosciences, University of Oxford, Oxford OX3 9DU, UK; Kavli Institute for Nanoscience Discovery, Oxford OX1 3QU, UK; Margarita Salas Center for Biological Research, University of Madrid, Madrid 28040, Spain; Nuffield Department of Clinical Neurosciences, University of Oxford, Oxford OX3 9DU, UK; Kavli Institute for Nanoscience Discovery, Oxford OX1 3QU, UK; Nuffield Department of Clinical Neurosciences, University of Oxford, Oxford OX3 9DU, UK; Kavli Institute for Nanoscience Discovery, Oxford OX1 3QU, UK; Nuffield Department of Clinical Neurosciences, University of Oxford, Oxford OX3 9DU, UK; Kavli Institute for Nanoscience Discovery, Oxford OX1 3QU, UK; Nuffield Department of Clinical Neurosciences, University of Oxford, Oxford OX3 9DU, UK; Kavli Institute for Nanoscience Discovery, Oxford OX1 3QU, UK; Department of Neurology, Klinikum Rechts der Isar, School of Medicine, Technical University of Munich, Munich 81675, Germany; Sir William Dunn School of Pathology, University of Oxford, Oxford OX1 3RE, UK; Nuffield Department of Clinical Neurosciences, University of Oxford, Oxford OX3 9DU, UK; Kavli Institute for Nanoscience Discovery, Oxford OX1 3QU, UK

**Keywords:** induced pluripotent stem cells, mitochondrial dysfunction, amyotrophic lateral sclerosis, motor proteins, axonal transport

## Abstract

Amyotrophic lateral sclerosis (ALS) is a neurodegenerative disease of the motor system with complex determinants, including genetic and non-genetic factors. A key pathological signature of ALS is the cytoplasmic mislocalization and aggregation of TDP-43 in affected motor neurons, which is found in 97% of cases. Recent reports have shown that mitochondrial dysfunction plays a significant role in motor neuron degeneration in ALS, and TDP-43 modulates several mitochondrial transcripts. In this study, we used induced pluripotent stem cell-derived motor neurons from ALS patients with TDP-43 mutations and a transgenic TDP-43^M337V^ mouse model to determine how TDP-43 mutations alter mitochondrial function and axonal transport. We detected significantly reduced mitochondrial respiration and ATP production in patient induced pluripotent stem cell-derived motor neurons, linked to an interaction between TDP-43^M337V^ with ATPB and COX5A. A downstream reduction in speed of retrograde axonal transport in patient induced pluripotent stem cell-derived motor neurons was detected, which correlated with downregulation of the motor protein complex, DCTN1/dynein. Overexpression of DCTN1 in patient induced pluripotent stem cell-derived motor neurons significantly increased the percentage of retrograde travelling mitochondria and reduced the percentage of stationary mitochondria. This study shows that ALS induced pluripotent stem cell-derived motor neurons with mutations in TDP-43 have deficiencies in essential mitochondrial functions with downstream effects on retrograde axonal transport, which can be partially rescued by DCTN1 overexpression.

## Teaser

Dafinca *et al.* show that mutations in TDP-43 lead to decreased mitochondrial oxidative phosphorylation, partially due to interactions with the ATP production machinery and COX5A. These have direct effects on axonal transport, which is reduced in amyotrophic lateral sclerosis motor neurons, and overexpression of dynactin-1 significantly increases retrograde mitochondrial dynamics.

## Graphical Abstract

**Graphical Abstract:**
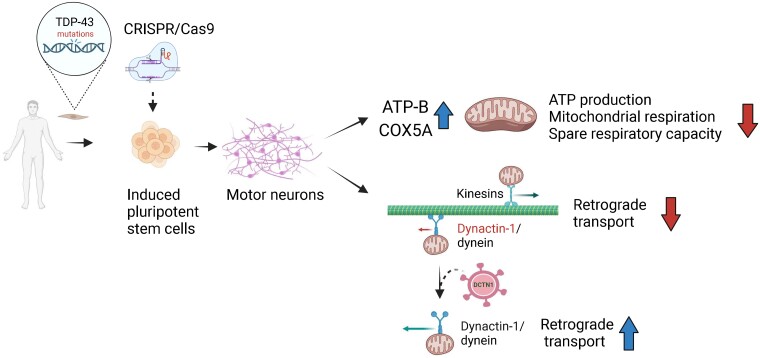

## Introduction

Amyotrophic lateral sclerosis (ALS) is a rapidly progressive neurodegenerative disorder of the motor system, characterized by loss of upper and lower motor neurons and profound muscle weakness. The pathological signature of ALS, present in 97% of cases, is the cytoplasmic mislocalization and aggregation of TDP-43.

Emerging evidence indicates that mitochondrial dysfunction contributes to the onset and progression of ALS. Several studies have demonstrated that TDP-43 accumulates in the mitochondrial fraction, and that preventing its translocation can rescue toxicity.^1-7^ Overexpression of mutant or wild-type TDP-43 in cultured motor neurons was shown to induce alterations in the morphology and transport of mitochondria reminiscent of observations in SOD1 mutant mice, along with aggregations of mitochondria in spinal cord motor neurons of mutant TDP-43 transgenic mice.^8,9^ Mitochondrial depolarization was also described in neuroblastoma cells and in primary motor neurons overexpressing mutant TDP-43^Q331K^ and TDP-43^M337V^.^8,10,11^ Furthermore, in TDP-43^G298S^ and TDP-43^A382T^ patient fibroblasts, complex I activity was decreased, along with reduced ATP levels and oxygen consumption.^2^ Since neurons require high levels of energy, it has been suggested that motor neurons may be significantly more vulnerable to mitochondrial dysfunction than other cell types.^12-14^

Axonal transport deficiency is a common mechanism that has been observed in many neurodegenerative disorders, including ALS.^15,16^ The transport of mitochondria, endosomes and vesicles containing trophic signalling receptors have all been reported to be affected in ALS models.^17-19^ The correct distribution of mitochondria in neurons is crucial to neuronal function, and this is supported by the fact that pathology in neurodegenerative diseases often correlates with defects in mitochondrial intracellular localization.^20-23^ Mitochondria provide the ATP necessary to actively transport mRNAs, proteins and organelles throughout the cells, in addition to its role in Ca^2+^ buffering and metabolite synthesis.^20^ Axonal transport of mitochondria is driven by ATP-driven molecular motors that carry organelles and vesicles along the microtubules and the main effectors are kinesins, mediating anterograde transport, and the major protein complex dynactin/dynein, mediating retrograde transport.^24^ The recruitment of dynactin-1 to the dynein complex is believed to be crucial for dynein function and for axonal transport,^25^ and mutations in its largest subunit (DCTN-1) have been reported in ALS patients.^26-29^

In this study, we show that mitochondrial bioenergetics are reduced in induced pluripotent stem cell-derived motor neurons (iPS-MNs) from TDP-43 patients carrying the M337V and I383T mutations and that these alterations are linked to a preferential interaction of mutant TDP-43 with mitochondrial targets involved in ATP production and mitochondrial respiration, ATP synthase and COX5A. These contribute to reduced neuronal survival and impaired speed of axonal transport. Furthermore, we show that ALS patient iPS-MNs have a downregulation of the heavy subunit of dynactin/dynein, DCTN-1, reducing the speed of retrograde mitochondrial transport in TDP-43^M337V^ and TDP-43^I383T^ iPS-MNs, and this impairment can be improved by overexpression of DCTN-1 in patient iPS-MNs.

## Materials and methods

### iPS cell generation and differentiation to MNs

All iPSC lines were derived from skin biopsy fibroblasts, collected under ethical approval granted by the South Wales Research Ethics Committee (WA/12/0186) in the James and Lillian Martin School Centre for Stem Cell Research, University of Oxford, under standardized protocols that we have described elsewhere.^30^ The demographics of patients and healthy controls (HCs) are described in Supplementary Table 1. Fibroblasts and derived iPSC lines tested negative for mycoplasma (MycoAlert, Lonza, UK). OXTDP-01 clones were derived using the Sendai virus-based reprogramming system CytoTune (Klf4, Oct4, Sox2 and c-Myc genes in individual viruses); OXTDP-03 clones were derived using CytoTune 2.0 (polycistronic vector Klf4–Oct3/4–Sox2, c-Myc and Klf4 separate viruses) (Life Technologies, Rockville, MD, used according to the manufacturer’s instructions). Transduced fibroblasts were plated for generation of iPSC clones, and clones were picked, expanded and banked as described previously.^31^ The iPS cells were differentiated to motor neurons *in vitro* according to our previously published methods.^30^ Briefly, the iPS cells were grown on Geltrex in mTESR™ 1 supplemented with mTESR™ supplement (Stem Cell Technologies) and antibiotics (Life Technologies). Induction was started when iPS cells were 90% confluent in DMEM/F-12/Neurobasal medium 1:1, 1× N2 supplement, 1× B27 supplement, ascorbic acid (0.5 μM, Sigma), β-mercaptoethanol (50 μM), compound C (1 μM) and Chirr99201 (3 μM, Tocris Bioscience). On Day 2, the induction medium was supplemented with all-trans retinoic acid (RA; 1 μM, Sigma) and Smoothened Agonist (SAG; 500 nM). On Day 4, we removed from the medium Chirr99201 and compound C. On Day 9, the neural precursors were split 1:3 using Accutase (Life Technologies), and Rock inhibitor (Tocris Bioscience) was added for 24 h. The neural precursors were maintained until Day 19 in this medium and changed to the basal medium supplemented with BDNF (10 μM, Life Technologies), GDNF (10 μM, Life Technologies) and laminin (500 ng/μL). DAPT (10 μM, Tocris Bioscience) was added for 7 days and withdrawn for the rest of the maturation. Analysis was performed between Days 30 and 35 of the differentiation protocol in all experiments.

### CRISPR/Cas9 correction of M337V mutation

TDP-43^M337V^ iPS cells from line TDP-43-03-03 were nucleofected (Neon, Life Technologies) with custom gRNAs, Alt-R Hi-Fi Cas 9 nuclease V3 (Integrated DNA Technologies), Alt-R CRISPR/Cas9 tracrRNA (IDT) and a single-strand DNA donor template Ultramer DNA Oligo (93 bp, IDT). Sequences can be found in Supplementary Table 2. After nucleofection, iPS cells were plated in mTESR^1^ supplemented with CloneR (Stem Cell Technologies) and allowed to grow until confluent. The pooled iPS cells were analysed by sequencing for efficiency of correction, and they were plated at low density on MEF (Life Technologies)-coated 6 cm dishes. Ninety-six single clones were picked into a 96-well plate in mTESR^1^, and genomic DNA was extracted for analysis using the manufacturer’s instructions (QIAquick Mini DNA Extraction kit). PCR products of the mutation region were sequenced for identifying corrected clones. The successfully corrected clones were analysed for pluripotency markers and karyotype integrity by previously described methods.^30^

### Mouse ES differentiation to MNs

Embryonic stem cells were obtained from a previously described transgenic mouse carrying the entire human genomic *TARDBP* gene with the M337V mutation and a C-terminal Ypet tag.^32^ mESCs were differentiated to motor neurons using a previously published protocol.^32^ In brief, mESCs were expanded on a layer of mouse primary embryonic fibroblasts (PMEFs; Thermo Fisher Scientific) in Knockout DMEM (Invitrogen) media, supplemented with 15% ESC-screened foetal bovine serum (Thermo Fisher Scientific), 2 mM L-glutamine (Invitrogen), 0.01% MEM non-essential amino acids (Invitrogen), 1 ng/mL leukaemia inhibitory factor (LIF), 0.01% EmbryoMax ESC qualified nucleosides (Millipore) and 0.1 mM 2-mercaptoethanol (Invitrogen). Following 3 days of incubation, embryoid bodies (EBs) were detached from the PMEF layer and dissociated into a single cell suspension by treatment with 0.25% trypsin-EDTA (Invitrogen). The resultant mESC suspension was plated into 10 cm dishes (Corning) in ADFNK media containing 50% advanced DMEM/F-12 (Invitrogen), 50% Neurobasal medium (Invitrogen), 10% Knockout serum replacement (Invitrogen), 2 mM L-glutamine (Invitrogen) and 0.1 mM 2-mercaptoethanol (Invitrogen). Following 2 days in ADFNK media, EBs were split up to 1:4 into new 10 cm dishes in ADFNK supplemented with 1 μM RA (Sigma) and 0.5 μM SAG (Merck) to promote a caudal identity. After three further days, EBs were collected and dissociated using Accumax (Sigma) and then seeded onto laminin-coated plates, incubated in ADFNK media supplemented with RA, SAG and neurotrophic growth factors (GDNF, BDNF, CNTF and NT-3, all at 10 ng/mL; PeproTech). Motor neurons were analysed 3 days after seeding.

### Immunostaining

Mature motor neurons were fixed with 4% paraformaldehyde–phosphate-buffered saline (PBS) for 15 min and incubated with 10% donkey serum in PBS with 0.2% Triton X for 1 h at room temperature. The cells were incubated overnight at 4°C with mouse or rabbit anti-β-III Tubulin (Tuj1) (Covance, 1:1000), mouse anti-TDP-43 (Proteintech, 1:500), rabbit anti-TDP-43 C-terminus (Proteintech, 1:1000), mouse anti-ATPB (Abcam, 1:500), rabbit anti-COX5A (Abcam, 1:500) and rabbit anti-DCTN-1 (Life Technologies, 1:500). After washing with 0.1% Triton X/PBS three times for 10 min, the samples were incubated with Alexa Fluor 488, Alexa Fluor 568 and Alexa Fluor 637 conjugated donkey anti-rabbit, anti-mouse or anti-goat secondary antibodies (Life Technologies) for 1 h at room temperature. Nuclei were stained with DAPI (Sigma-Aldrich). Fluorescence was visualized using a Spinning Disc Confocal Microscope Zeiss and an Airyscan LSM 910 (Micron Bioimaging Facility, Oxford).

### Immunoblotting

Cells were lysed in RIPA buffer (Life Technologies), sonicated and incubated on ice. After centrifugation at 6000*×g* for 10 min, the supernatant was retained and protein concentration was quantified using BCA assay (Sigma). Protein was loaded and resolved on a SDS-PAGE (4–12% Tris–glycine gel) using a Mini-Protean electrophoresis system (Life Technologies) under constant current and transferred to a polyvinylidene difluoride (PVDF) membrane (Immobilon-P) using the iBlot2 system Novex Blot system. Blots were transferred to blocking buffer (TBS, 0.1% Tween-20, 5% skimmed milk) for 1 h at room temperature and incubated in TBS + 0.1% Tween-20 + 1% milk with primary antibodies. Primary antibodies used were mouse anti-ATPB (Abcam, 1:1000), mouse anti-COX5A (Abcam, 1:500), rabbit anti-Dynactin-1 (Life Technologies, 1:500), mouse anti-β-actin (Sigma, 1:5000), mouse anti-GAPDH (Abcam, 1:1000), mouse anti-Dynein (Thermo Fisher Scientific, 1:500), rabbit anti-KIF5A (Abcam, 1:500), rabbit anti-KIF5B (Abcam, 1:500), rabbit anti-KIF5C (Abcam, 1:500), rabbit anti-MFN1 (Cell Signaling, 1:500), mouse anti-OPA1 (Sigma, 1:500) and rabbit anti-MFN2 (Cell Signaling, 1:500). Horseradish peroxidase-conjugated anti-mouse IgG or anti-rabbit IgG (Life Technologies) were used as secondary antibodies, and the signal was visualized using an ECL or ECL Plus detection system (Millipore) on a Bio-Rad ChemiDoc. The integrated optical density of each band was measured in FIJI, and expression was normalized to β-actin or GAPDH levels in the same blot for comparative expression assessment.

### Immunoprecipitation

Immunoprecipitation was performed on differentiated iPS-MNs on Day 35, according to the manufacturer’s instructions (Co-IP Dynabeads, Thermo Fisher). Briefly, neuron plates on 10 cm dishes were washed with PBS and lysed in Pierce IP lysis buffer, supplemented with cOmplete EDTA-free protease inhibitor cocktail (Roche). Protein concentration was assessed by standard BCA, performed according to the manufacturer’s instructions (Pierce). The same amount of protein was prepared from all samples in 200 µL; 1.5 mg Dynabeads per sample were washed in C1 buffer and were coupled with either 12 µg Rabbit IgG antibody (Abcam) or 12 µg anti-TDP-43 antibody (rabbit, Abcam) overnight at 4°C. The conjugated Dynabeads were separated on a DynaMag (Invitrogen) magnet the next day and washed with HB, LB and SB buffers. For the final wash, the Dynabeads were rinsed in IP lysis buffer, followed by incubation of the protein samples overnight, on a rotator, at 4°C with either IgG or TDP-43 antibody-coupled Dynabeads. The next day, the samples were removed from the rotator and placed on a DynaMag to wash with IP lysis buffer twice, followed by a wash on rotator for 5 min in LWB buffer with added 0.02% Tween-20. The bead suspension was transferred to a fresh tube, and the beads allowed to gather on the DynaMag. Elution was done using 100 µL EB buffer, on a rotator for 10 min, followed by removing the supernatant containing the IP complexes in a fresh tube.

### Mitochondrial transport

Motor neurons grown in Xona MicroChips (Xona) were incubated for 20 min with 100 nM MitoTracker Green FM (Life Technologies) at 37°C. The neurons were briefly washed three times with Live Imaging Solution (Life Technologies) and imaged on a Scanning Disc Confocal Zeiss Microscope. Time-lapse 3D images were captured every 1 s for 5 min as five-layer z-stacks. These settings were experimentally determined based on considerations of time resolution, tracking duration and photo-bleaching. Data were analysed using FIJI and a previously published MATLAB toolbox for the detection of mitochondrial movement in microfluidic chambers, MitoQuant.^33^ Live Imaging Solution containing 100 µM glutamate was added to the chamber wells, allowed to diffuse for 1 min, and axonal transport was then imaged as described above. Speed was measured only in mitochondria that were continuously moving, and any mitochondrion that was moving <0.1 um/s was not included in the speed analysis. The analysis consists of two image analysis programmes: MiTracker, for tracking mitochondria and linking their coordinates into 3D, and the motion pattern analyser, for identifying moving mitochondria in a 2D space, allowing measurement of transient and sustained speeds. The states of dynamic pause and both running states (anterograde and retrograde) are defined as motile or active mitochondrial states. In the ‘dynamic pause’ state, mitochondria exhibit fast switching between directions of anterograde and retrograde, which result in oscillatory motion with no net displacement. The dynamic pause is much shorter (<15 s) than the stationary state and has a higher probability to transition to running states. In both the anterograde and retrograde states, mitochondrial movement is characterized by ‘smooth and steady runs’ at a constant velocity. The stationary mitochondrial state was identified as those points located at the origin of coordinates zero. Data were collected from 3 independent microgrooves for each iPS line, recording between 200 and 900 mitochondria per data point.

### Endosomal transport

Motor neurons grown in Xona MicroChips (Xona) were incubated for 30 min with cholera toxin subunit B (CTB) labelled with Alexa Fluor 488 at 200 ng/mL (Thermo Fischer Scientific) at 37°C. The neurons were briefly washed three times with Live Imaging Solution (Life Technologies) and imaged on a Scanning Disc Confocal Zeiss Microscope. Time-lapse 3D images were captured every 1 s for 5 min. Data were analysed using the TrackMate plugin from ImageJ. Three independent differentiations were analysed, and three iPSC lines were pooled for TDP-43^M337V^ and two iPSC lines were pooled for TDP-43^I383T^.

### Transmission electron microscopy

Motor neurons on coverslips were fixed in pre-warmed 2.5% glutaraldehyde + 4% PFA in 0.1 M PIPES buffer at pH 7.2 for 1 h at room temperature, then moved to the fridge overnight. Samples were then washed in 0.1 M PIPES (5 changes of fresh buffer, 5–10 min each), followed by 15 min in 50 mM glycine in 0.1 M PIPES, then one last 10 min wash in 0.1 M PIPES. For the secondary fixation, samples were incubated in 1% osmium tetroxide + 1.5% potassium ferrocyanide in 0.1 M PIPES buffer at 4°C for 1 h and washed with MQ water. For the tertiary fixation, samples were incubated in 0.5% uranyl acetate overnight at 4°C in the dark. The neurons were then rinsed with water for 3 × 10 min and dehydrated by incubation in ice-cold 30, 50, 70, 80, 90 and 95% ethanol, each for 10 min. The samples were incubated in 100% dry ethanol for 20 min, and then the procedure was repeated twice. Epoxy resin infiltration was performed with Agar 100 resin. The samples were incubated with 3:1 100% dry ethanol:resin for 1 h, then 1:1 100% dry ethanol:resin for 2 h and 1:3 100% dry ethanol:resin for 1 h in the fume hood with rotation. Then the samples were incubated in 100% resin overnight at RT, and the resin was changed twice the next day. For embedding, the coverslips were inverted onto Beem capsules filled with fresh 100% resin. Agarose pieces were transferred into Beem capsules filled with fresh resin. Blocks were polymerized for 24 h in a 60°C oven. Polymerized locks were submerged in liquid nitrogen, and then the coverslip was snapped off, leaving the cells polymerized as a monolayer on the top surface of the block.

Ultrathin sections (90 nm) were taken using a Diatome diamond knife on a Leica UC7 ultramicrotome and mounted onto 200-mesh Cu grids or formvar Cu slot grids.

The grids were post-stained for 5 min with Reynold’s lead citrate, washed with degassed water, air dried and then imaged using a Thermo Fisher Tecnai T12 TEM operated at 120 kV using a Gatan OneView camera.

### Lentiviral DCTN-1-mGFP transduction

Lentiviral particles carrying human-tagged ORF clone DCTN1-mGFP (variant 2; NM_023019) and lenti ORF control particles of pLenti-C-mGFP-P2A-Puro (PS100093V) were purchased from OriGene. The optimal multiplicity of infection was determined as MOI = 5, and consequently, all microfluidic chambers were transduced for 18 h.

### RNA sequencing

RNA sequencing on HC and TDP-43 iPS-MNs was performed and analysed as previously described.^30^

### Interactome in mouse ES-MNs

The interactome of TDP-43-Ypet (WT) and TDP-43-Ypet (M337V) in mouse ES-MNs was previously described.^34^

### Statistical analysis

Data are represented as mean ± SD and analysed by one- or two-way ANOVA followed by a Sidak, Bonferroni, Tukey or Dunnett’s multiple comparison test. Data points represent replicates of individual iPS lines (three iPS lines for TDP-43^M337V^, two iPS lines for TDP-43^I383T^ and two iPS lines for HCs). A *P* < 0.05 was considered significant. GraphPad Prism was used for statistical analysis and to generate graphs. Co-localization analysis was performed using the Co-Localization plugin in ZEN Blue software (Zeiss) and JACoP plugin in Fiji. The MATLAB plugin MitoQuant was used to statistically analyse mitochondrial movement.

## Results

### ATP production and basal respiration are reduced in TDP-43^M337V^ and TDP-43^I383T^ iPS-MNs

**Figure 1:**
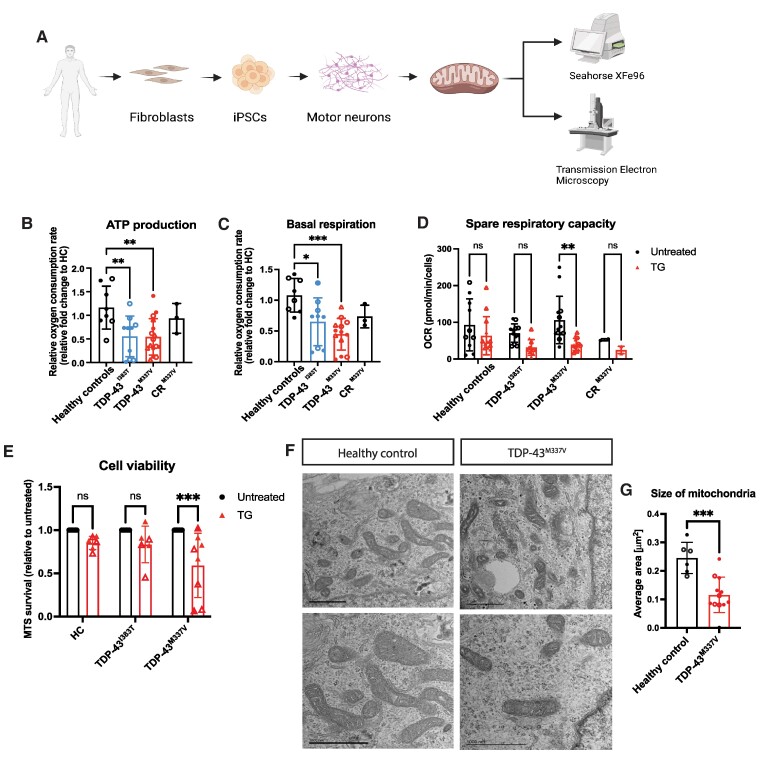
**Basal respiration and ATP production are reduced in iPS-MNs with TDP-43 mutations.** (**A**) Schematic representation of the experimental design. **(B**) ATP production is lower in TDP-43^M337V^ (**P = 0.004) and TDP-43^I383T^ iPS-MNs (**P = 0.010); *n* = 4 independent differentiations for HC lines OX3-8, 841 (HCs) and TDP-43^M337V^ and *n* = 3 for TDP-43^I383T^, one-way ANOVA with Dunnett’s *post hoc*, *F* = 4.61. (**C**) Basal respiration is significantly reduced in TDP-43^M337V^ (***P = 0.002) and TDP-43^I383T^ MNs (*P = 0.036); one-way ANOVA with Dunnett’s *post hoc*, *F* = 4.83. (**D**) TDP-43^M337V^ iPS-MNs have lower spare respiratory capacity after 1 μM thapsigargin (**P = 0.0011, *n* = 3 independent differentiations); two-way ANOVA with Sidak’s *post hoc*, *F* = 9.72. Data are represented as mean ± SD; *n* = 2 independent differentiations for HCs and TDP-43^M337V^, 3 independent iPS lines; *n* = 1 for TDP-43^I383T^ with 2 independent iPS lines. Data points represent individual iPS lines from each differentiation (ns = non-significant). (**E**) Neuronal viability was assessed by cell proliferation test and showed a significant reduction in survival of TDP-43^M337V^ iPS-MNs after 24 h treatment with TG (***P = 0.0003, two-way ANOVA with Tukey’s *post hoc*) (*n* = 3 independent differentiations). (**F**, **G**) TEM of mitochondria in HC (841) and 2 iPS lines from TDP-43^M337V^ shows reduced area for mutant TDP-43 mitochondria (***P = 0.0005, *F* = 1.291, Student’s *t*-test, *n* = 2 independent differentiations; data points represent average measurements from one field of view with 3–11 mitochondria, with at least 3 fields of view analysed per iPS line). Scale bars: top 1 μm, bottom 1000 nm.

Mitochondria play a key role in energy metabolism, acting as a source of energy for the majority of cellular processes. Neurons generate ATP by mitochondrial (oxidative phosphorylation) and non-mitochondrial (glycolysis) metabolism. To study mitochondrial bioenergetics in human iPS-MNs, we used a Seahorse Extracellular Flux Analyzer to monitor cellular oxygen consumption (OCR) in real time as measures of mitochondrial respiration (Fig. 1A). To isolate the effects of the mutation in iPS-MNs, we also generated a CRISPR/Cas9 correction of the M337V mutation (CR^M337V^) and confirmed its pluripotency and normal karyotype, as well as comparable motor neuron differentiation efficiency (Supplementary Figs 1 and 2). In TDP-43 mutant iPS-MNs, we found that ATP production was reduced by 50% compared to HCs (Fig. 1B). Basal respiration was also found to be significantly reduced, up to half in TDP-43^M337V^ and TDP-43^I383T^ compared to HCs, without significant differences in maximal respiration or spare respiratory capacity at baseline (Fig. 1C, Supplementary Fig. 3).

Mitochondria are in close physical contact with the endoplasmic reticulum (ER) and functionally connected. In the presence of ER stress, activation of mitochondrial respiration has been shown to be critical in mediating the cellular stress response and to promote survival.^35,36^ To test mitochondrial metabolic response to ER stress conditions, we treated iPS-MNs with 1 µM thapsigargin, which blocks the SERCA pump on the ER membrane, for 24 h, and we detected a significant reduction in spare respiratory capacity in TDP-43^M337V^, while no differences were detected in the HCs, CR^M337V^ or TDP-43^I383T^ (Fig. 1D). These reductions in mitochondrial metabolism also correlated with a significant reduction in cell survival in TDP-43^M337V^ after TG treatment (Fig. 1E). Using transmission electron microscopy (TEM), we identified that mitochondria in TDP-43^M337V^ have some morphological differences, with mitochondria appearing smaller (0.153 μm^2^) than HCs (0.236 μm^2^) (Fig. 1F and G). To check whether these differences in size were attributable to impaired fusion/fission events, we assessed levels of MFN2 and pDrp1 and confirmed that there were no differences in these markers (Supplementary Figs 4 and 15).

These results indicate that mitochondrial function and structure are impaired by TDP-43 mutations, and the M337V mutation renders motor neurons more susceptible to cell death under stress conditions.

### Mutant TDP-43^M337V^ alters expression of ATPB and COX5A in human and mouse MNs

**Figure 2:**
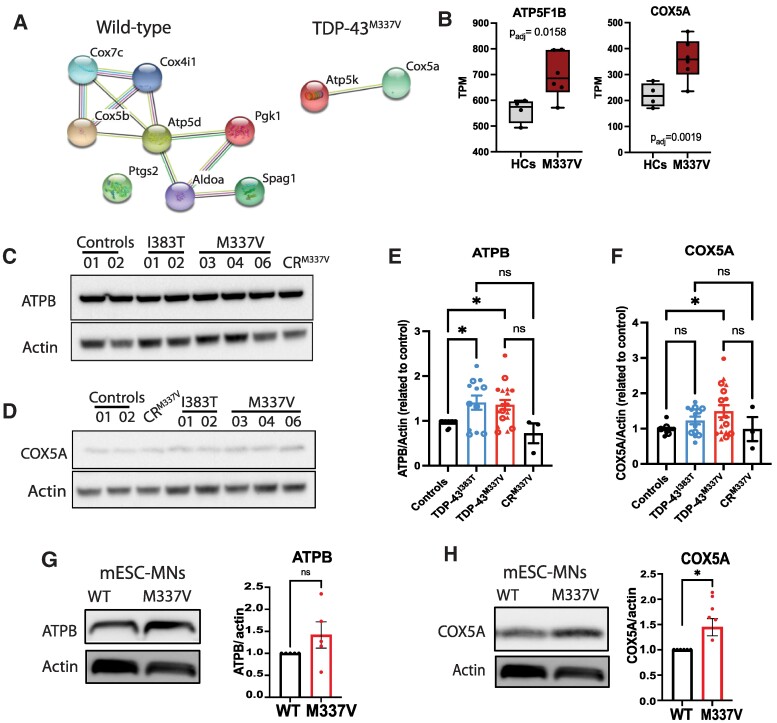
**Mutant TDP-43^M337V^ increases expression of ATP synthase and COX5A and co-localizes with ATPB in human iPS-MNs.** (**A**) Interactome targets of TDP-43^WT^ and TDP-43^M337V^ involved in ATP metabolism identified in mouse embryonic stem cell (ES)-MNs (Atp5k and Cox5a). (**B**) Normalized expression of ATP5B1F and COX5A (transcripts per million, TPMs) in RNA sequencing from iPS-MNs shows these transcripts are significantly increased in TDP-43^M337V^ iPS-MNs (*ATP5F1B P* = 0.0158; *COX5A P* = 0.0019, significant figures derived from DESeq2 analysis; data points represent technical replicates; *n* = 3 independent differentiations). (**C**, **D**) Immunoblotting of ATPB and COX5A in human iPS-MNs shows (**E**) increased levels of ATPB in TDP-43^M337V^ (*P = 0.030) and TDP-43^I383T^ iPS-MNs (*P = 0.026) compared to HCs (*n* = 5 independent differentiations, one-way ANOVA with Bonferroni *post hoc*, *F* = 4.82) and (**F**) increased levels of COX5A in TDP-43^M337V^ (*P = 0.019, *n* = 5 independent differentiations, one-way ANOVA with Bonferroni *post hoc*, *F* = 2.94). Symbols represent individual iPS lines, and each data point represents a technical replicate (between 1 and 3 technical replicates per differentiation; ns = non-significant). (**G**, **H**) Immunoblotting confirms high levels of *Cox5a* in mouse ES-derived MNs from TDP-43^M337V^-Ypet compared to wild-type (WT) (*n* = 3 independent differentiations, one-way ANOVA with Dunnett’s *post hoc*). Data are presented as mean ± SD. See Supplementary Fig. 10 for uncropped blots.

In a recent interactome study, we identified that different interactors were co-immunoprecipitated with TDP-43^WT^ or TDP-43^M337V^ in motor neurons derived from embryonic stem cells of a BAC transgenic mouse model of TDP-43.^34^ Despite an overall reduction of interactors, including mitochondrial proteins, with TDP-43^M337V^ (*n* = 43 interactors) compared to TDP-43^WT^ (*n* = 249 interactors), two members of the mitochondrial electron transfer chain (Atp5k and Cox5a) were acquired more abundantly by mutant TDP-43, but not by wild-type TDP-43 (Fig. 2A). Furthermore, RNA sequencing performed on iPS-MNs from TDP-43^M337V^ and TDP-43^I383T^ patients has shown an upregulation of constituents of ATP metabolism and the mitochondrial electron transfer chain (ATPB and COX5A), indicating mutant TDP-43 may have both a direct and indirect effect on the expression of members of the mitochondrial electron transfer chain complex (Fig. 2B). To confirm these transcriptional changes, we analysed the expression of ATPB (*ATP5BF1*) and COX5A in both iPS-MNs and in mouse ES-MNs. In TDP-43^M337V^ iPS-MNs, we detected increased protein expression of ATPB and COX5A compared to HCs (Fig. 2C–F, Supplementary Fig. 10). Analysis of ES-derived MNs from the transgenic TDP-43^M337V^-Ypet mouse did not show a significant increase in ATPB (Fig. 2G), but also confirmed a significant upregulation of Cox5A compared to the transgenic TDP-43^WT^ (Fig. 2H). These results show that motor neurons carrying the M337V mutation in TDP-43 upregulate expression of ATPB and COX5A, both at transcriptional and protein levels in human iPS-MNs, while the I383T mutation in TDP-43 upregulates only COX5A transcriptional expression.

### Mutant TDP-43^M337V^ co-localizes with subunits of ATP synthase and COX5A in patient iPS-MNs

**Figure 3:**
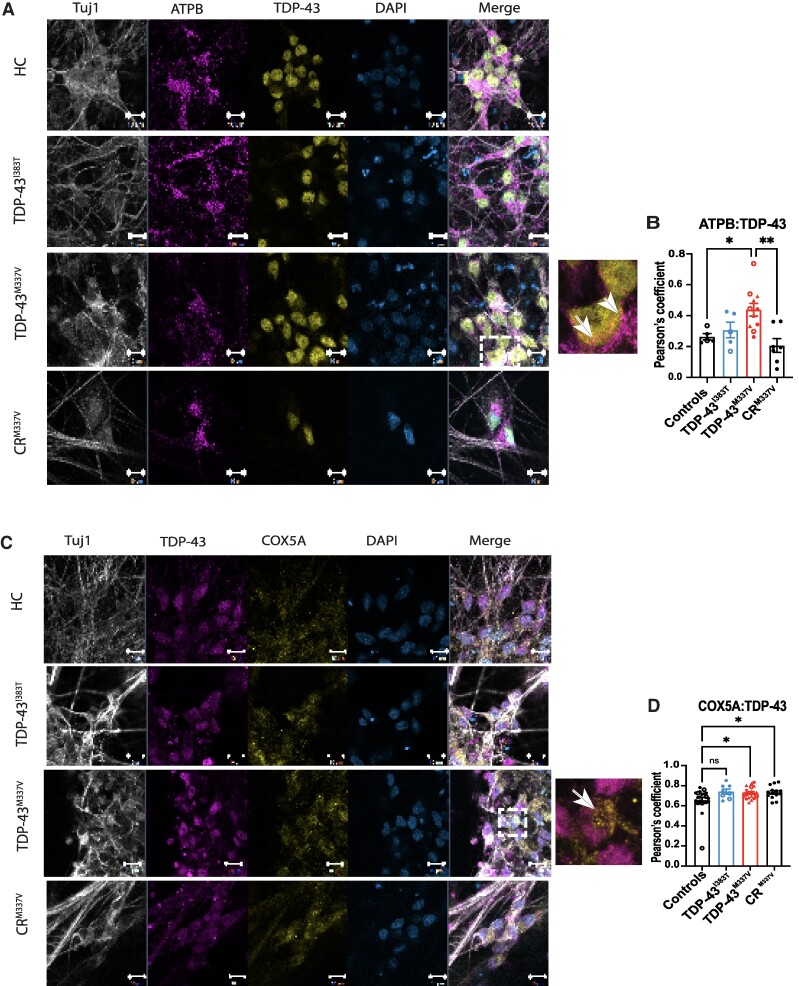
**Mutant TDP-43^M337V^ co-localizes with ATPB and COX5A in human iPS-MNs.** (**A**, **B**) Increased co-localization of TDP-43 with ATP synthase subunit b (ATPB) is detected in TDP-43^M337V^ iPS-MNs compared to HCs (*P = 0.041) and CR^M337V^ (**P = 0.001) (*n* = 3 independent differentiations, 2 independent clones for I383T and 3 independent clones for M337V). (**C**, **D**) Increased co-localization with COX5A is detected in TDP-43^M337V^ (*P = 0.0316) and CR^M337V^ (*P = 0.0388, *n* = 3 independent differentiations, 3 clones for M337V and 2 clones for I383T). One-way ANOVA with Dunnett’s *post hoc* test. Symbols represent individual iPS lines, and each data point represents a technical replicate (1–3 technical replicates per differentiation; ns = non-significant). Data are presented as mean ± SD. Scale bar = 10 μm.

To investigate if mutant TDP-43 interacts directly with members of the ATP synthase complex and electron transfer chain, we performed co-localization experiments. In human iPS-MNs, we detected a significantly increased co-localization between TDP-43^M337V^ and the beta subunit of ATP synthase, ATPB, which was corrected in the CR^M337V^ (Fig. 3A and B). Furthermore, we also confirmed an increased co-localization between TDP-43 and COX5A in human iPS-MNs carrying the M337V mutation, which was not rescued by CRISPR correction of the mutation in CR^M337V^ (Fig. 3C and D). However, by immunoprecipitation of endogenous TDP-43, we did not observe a pull down of these mitochondrial membrane proteins (Supplementary Figs 5A and 12A and B). We also confirmed that these interactions were not due to increased cytoplasmic TDP-43 or increased total TDP-43 expression in patient lines (Supplementary Fig. 6A and B). Furthermore, we do not observe co-localization or co-immunoprecipitation between TDP-43 and TOMM20, indicating there may be a specific interaction within the inner mitochondrial membrane (Supplementary Figs 6C and D and 12C). In mouse ES-derived MNs, we did not detect a significantly increased co-localization between ATPB and TDP-43^M337V^ (Supplementary Fig. 7A and B).

In order to determine if the interaction was due to increased levels of mutant TDP-43 translocated to the mitochondria, we investigated the protein levels of TDP-43 in the mitochondria of HCs and mutant iPS-MNs. Mitochondria were extracted from HCs, TDP-43^M337V^ and TDP-43^I383T^ iPS-MNs and analysed by immunoblotting along with the cytosolic fractions (Supplementary Figs 8A and 13). We detected no significant differences between genotypes in the expression of full-length TDP-43 and 35 kDa TDP-43 at baseline, after ER stress (TG) and proteasomal inhibition (MG-132) (Supplementary Fig. 8B–D).

These results show that mutant TDP-43^M337V^ may interact indirectly with ATP synthase and COX5A specifically in human iPS-MNs, where it may contribute to altering their function, leading to a feedback mechanism of increased protein expression.

### Retrograde axonal transport speed is reduced in TDP-43 iPS-MNs

Deficiencies in axonal transport have been identified as an early phenotype in ALS models. ATP production is essential for efficient axonal transport in neurons, and a previous study has also shown reduction in mitochondrial movement in C9OFR72 iPS-MNs due to low mitochondrial bioenergetics.^37^ Since mitochondrial ATP production is lowered in TDP-43 patient iPS-MNs and we found mutant TDP-43 co-localizing to mitochondrial ATP synthase, we aimed to determine if axonal transport was reduced as a consequence.

**Figure 4:**
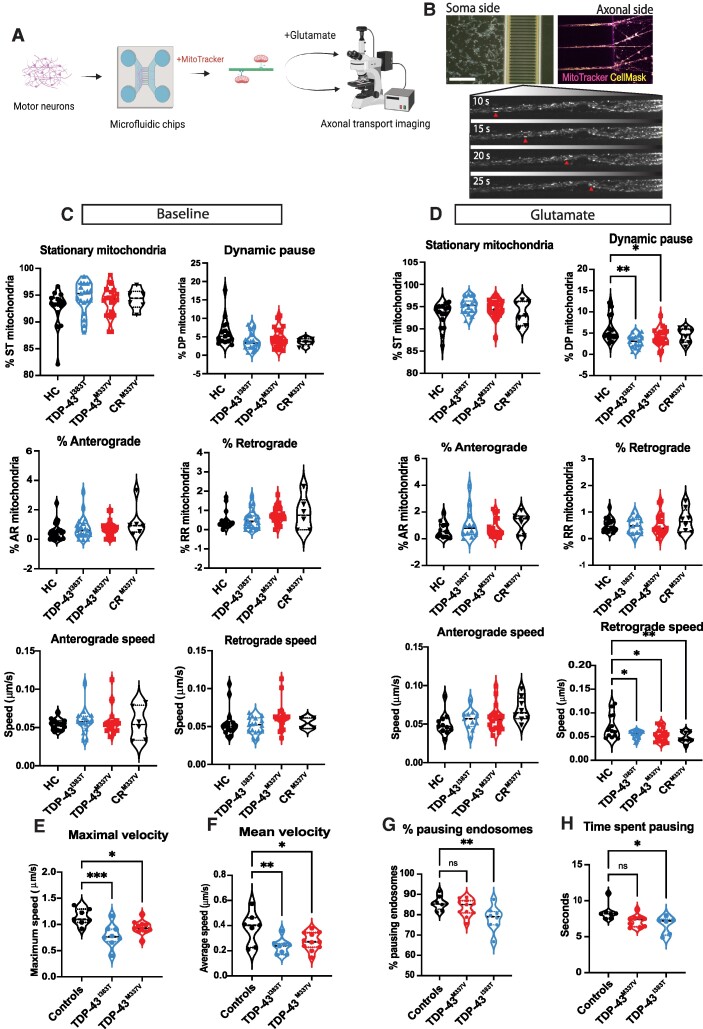
**Retrograde mitochondrial and endosomal transport in TDP-43 MNs is slower.** (**A**) Experimental design of the axonal transport analysis. (**B**) iPS-MNs in microfluidic chambers stained with MitoTracker (scale bar = 450 µm). (**C**) Analysis of axonal transport of mitochondria at baseline shows no differences in percentage of stationary mitochondria (ST), dynamic pause mitochondria (DP), anterograde (AR), retrograde (RR) mitochondria or in their speeds (one-way ANOVA, ns). (**D**) Analysis of mitochondrial transport in axons after stimulation with 100 μM glutamate shows significantly lower percentage of mitochondria in dynamic pause (*P* = 0.0013) in TDP-43^I383T^ iPS-MNs and TDP-43^M337V^ (*P = 0.0370, *F* = 4.76 one-way ANOVA with Dunnett’s *post hoc*) compared to HCs; no significant differences were detected in percentage of anterograde and retrograde running mitochondria; the speed of retrograde mitochondria in TDP-43^M337V^, TDP-43^I383T^ and CR^M337V^ is significantly lower than the HC (*P = 0.011, *P = 0.022 and **P = 0.006, respectively). Each data point represents an average value from 200 to 800 mitochondria obtained from an individual recording of 5 min from each iPS-MN line (*n* = 4 independent differentiations with at least 3 independent recordings from microgrooves; 2–3 iPS lines are pooled for TDP-43^M337V^ and 2 iPS lines are pooled for TDP-43^I383T^, one-way ANOVA followed by Dunnett’s *post hoc* test, *F* = 4.61). (**E**) Maximal endosomal velocity is significantly reduced in TDP-43^M337V^ (*P = 0.033) TDP-43^I383T^ iPS-MNs (***P = 0.0008). (**F**) Reduced mean velocity is detected in TDP-43^M337V^ (*P = 0.033) and TDP-43^I383T^ (**P = 0.006) iPS-MNs (*n* = 3 independent differentiations). (**G**) Endosomes pause more frequently in HCs than in TDP-43^I383T^ (**P = 0.005). (**H**) The time spent pausing is longer (*P = 0.017) in HCs, but no differences are detected between controls and TDP-43^M337V^ (ns = non-significant). Data points represent average values obtained from each recording (one-way ANOVA with Sidak’s multiple comparison test).

Motor neurons were differentiated in microfluidic chambers, and axons were guided through the microgrooves by enhancing the medium with 10× BDNF and GDNF. Using MitoTracker Green FM, the movement of mitochondria was visualized (Fig. 4A). Mitochondrial movement, speed and direction were quantified at baseline and after 1 min stimulation with 100 µM glutamate (Fig. 4B). Using these conditions, we previously reported calcium buffering deficits in our iPS-derived MNs from C9ORF72 and TDP-43 mutation carriers.^30^ In basal conditions, there were no differences detected between HCs, CRISPR/Cas9-corrected isogenic control and TDP-43 patient iPS-MNs in the percentage of stationary mitochondria, anterograde and retrograde running mitochondria, mitochondria in dynamic pause or speeds of anterograde and retrograde mitochondria (Fig. 4C). In the presence of glutamate, we detected significantly fewer mitochondria in dynamic pause (4.13 and 3.09% for TDP-43^M337V^ and TDP-43^I383T^, respectively) compared to HCs (6%) (Fig. 4D). While there were differences in the percentage of anterograde and retrograde running mitochondria among the genotypes, both TDP-43^M337V^ (0.054 µm/s) and TDP-43^I383T^ (0.054 µm/s) mitochondria travelled retrogradely at significantly reduced speeds compared to HCs (0.071 µm/s). The correction of the M337V mutation did not rescue this phenotype, and the mitochondria in CR^M337V^ also showed a significantly reduced speed compared to HCs (0.048 µm/s).

In addition to mitochondrial transport, we also analysed endosomal transport in motor neurons differentiated in microfluidic chambers. Neurons were loaded with CTB, which is actively recruited for retrograde transport by the endosomes. In TDP-43^M337V^ and TDP-43^I383T^, we found that maximal velocity was significantly reduced at 0.94 and 0.78 µm/s, respectively, compared to 1.1 µm/s in HCs (Fig. 4E). Similarly, analysis of mean velocity showed a significant reduction in retrograde endosomal speed with both mutant lines (0.27 and 0.23 µm/s, respectively) compared to HCs (0.38 µm/s) (Fig. 4F). These results are in line with previous findings in our TDP-43^M337V^ transgenic mouse model, where a reduction in endosomal transport was reported *in vivo*.^38^

In order to analyse whether the overall reduction in speed may be due to higher frequency of pausing endosomes, rather than continuous speed, we assessed the time endosomes spent pausing, as well as the percentage of pausing endosomes for each patient and controls (Fig. 4G and H). The results show that the controls have the highest percentage of pausing endosomes (85.7%) compared to TDP-43^M337V^ (83.5%) and TDP-43^I383T^ (78.1%) and spend an average of 8.4 s in pause, compared to 7.2 s for TDP-43^M337V^ and 6.6 s for TDP-43^I383T^ endosomes (Fig. 4G and H).

These results confirm that mutations in TDP-43 contribute to a reduction in retrograde axonal transport that affects both mitochondria and endosomes.

### Motor proteins involved in axonal transport are differentially expressed in TDP-43 iPS-MNs

The transport of mitochondria and endosomes along axons is tightly regulated by motor proteins that are specific for each direction of movement along the microtubules. Cytoplasmic dynein is a multi-subunit protein complex, with heavy chains having ATPase activity and interacting with light chains to generate the cargo-binding complex and binds to the microtubules to mediate retrograde transport.^39,40^ To achieve its full functional role, dynein requires dynactin, mutations in the largest subunit (dynactin-1 or DCTN-1) of which were previously linked to ALS.^41^

**Figure 5:**
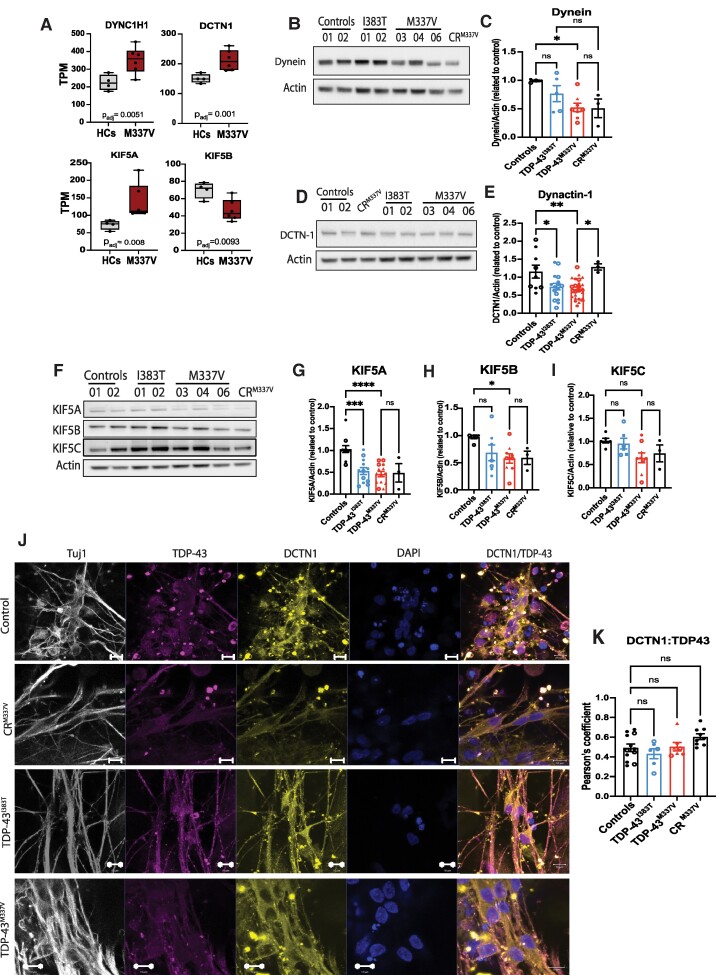
**Motor proteins are differentially expressed in TDP-43 MNs.** (**A**) RNA sequencing shows significant differential expression of Dynein, DCTN1, KIF5A and KIF5B in TDP-43^M337V^ compared to HCs. Each data point represents an individual iPS line (*n* = 3 independent differentiations). (**B**, **C**) Immunoblotting shows Dynein is significantly downregulated in TDP-43^M337V^ (*P = 0.036, *F* = 2.047) (one-way ANOVA with Sidak’s *post hoc*). (**D**, **E**) Western blotting confirms a significant decrease of DCTN1 in patient MNs TDP-43^I383T^ (*P = 0.029) and TDP-43^M337V^ (**P = 0.004) (*n* = 8 independent differentiations for TDP-43^I383T^ and TDP-43^M337V^; *n* = 5 independent differentiations for HCs; *n* = 3 differentiations for CR^M337V^; each data point represents an individual iPS line from each differentiation; symbols represent the different iPS lines used for each patient or control line). A significant increase is detected in CR^M337V^ compared to TDP-43^M337V^ (*P = 0.027). Data are represented as mean ± SD. One-way ANOVA with Sidak’s *post hoc* test (*F* = 2.92). (**F**) Western blotting of KIF5A, KIF5B and KIF5C shows that (**G**) KIF5A is downregulated in TDP-43^M337V^ (***P = 0.0005, *F* = 11.53) and TDP-43^I383T^ (****P < 0.001) and (**H**) KIF5B is significantly downregulated in TDP-43^M337V^ (*P = 0.0299, *F* = 2.78) (one-way ANOVA with Sidak’s *post hoc*). (**I**) KIF5C is not differentially expressed in TDP-43 iPS-MNs (ns). (**J**, **K**) TDP-43^M337V^ and TDP-43^I383T^ do not preferentially co-localize with DCTN1 in patient MNs (*n* = 3 independent differentiations, one-way ANOVA with Sidak’s *post hoc;* ns = non-significant). Scale bar = 10 µm. See Supplementary Fig. 11 for uncropped blots.

Our RNA sequencing data showed that several motor proteins were differentially expressed in TDP-43^M337V^ iPS-MNs compared to HCs (Fig. 5A). By immunoblotting, we confirmed a downregulation of dynein in TDP-43^M337V^ at the protein level (Fig. 5B and C). We also found that dynactin-1 protein was significantly downregulated in both TDP-43^M337V^ and TDP-43^I383T^ compared to HCs, and the levels were normalized by correcting the M337V mutation in CR^M337V^ (Fig. 5D and E). Furthermore, two anterograde motor proteins, KIF5A and KIF5B, were also significantly downregulated in the TDP-43^M337V^ line, while KIF5C levels were normal in all iPS-MNs (Fig. 5F–I, Supplementary Fig. 11).

To determine if TDP-43 directly interacts with DCTN-1, we performed co-localization and immunoprecipitation experiments (Fig. 5J). By immunostaining, we did not detect a significantly increased co-localization between TDP-43 and DCTN-1, even under stress conditions (Fig. 5K, Supplementary Fig. 8A). Furthermore, to assess a direct binding of TDP-43 with the retrograde motor proteins, dynein and DCTN1, we performed immunoprecipitations of endogenous TDP-43, which confirmed that TDP-43 does not directly bind to the complex (Supplementary Figs 9B and 14).

These results indicate that mutant TDP-43 contributes to the reduction in retrograde transport through an indirect downregulation in the expression of anterograde/retrograde proteins and by reducing mitochondrial bioenergetics, and not through a direct interaction with the motor proteins involved in retrograde transport.

### Overexpression of dynactin-1 increases retrograde transport

**Figure 6:**
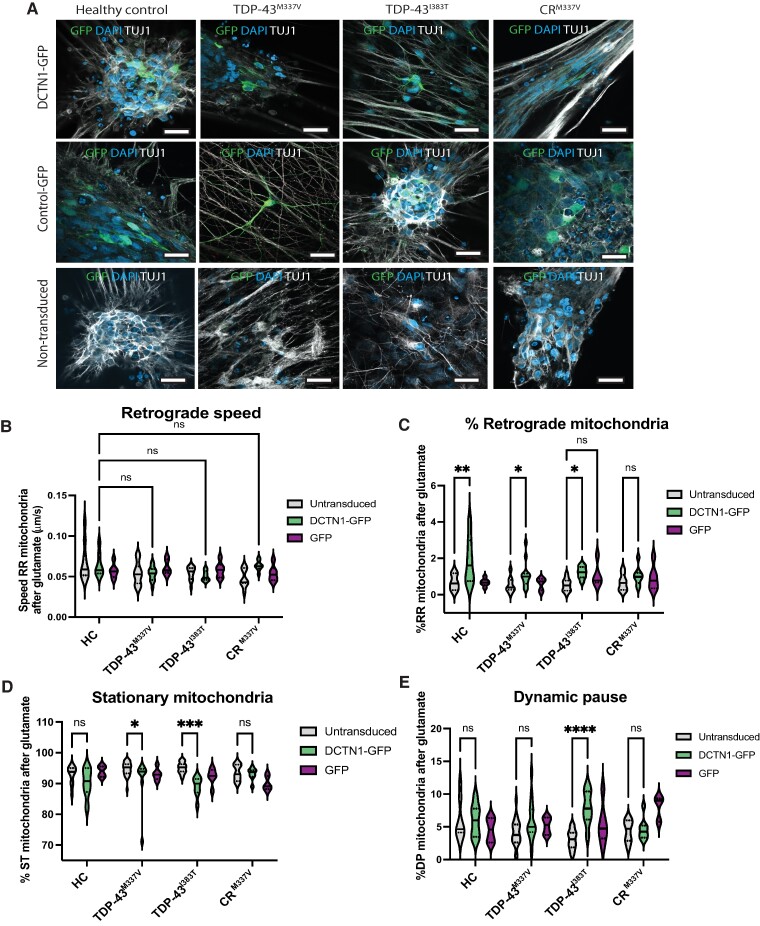
**Lentiviral DCTN1 overexpression increases axonal transport in TDP-43 iPS-MNs.** (**A**) Representative images of lentiviral expression of DCTN1-GFP and control-GFP in HCs, TDP-43^M337V^, TDP-43^I383T^ and CR^M337V^ iPS-MNs, as well as non-transduced neurons. Neurons are stained for Tuj1 and nuclei are labelled by DAPI. Scale bar = 20 µm. (**B**) Speed of retrograde mitochondria is not different between HCs and patient iPS-MNs after transduction with DCTN1-GFP (ns, non-significant) (two-way ANOVA with Sidak’s *post hoc*). (**C**) The percentage of retrograde running mitochondria is significantly increased by DCTN1 expression in all iPS-MNs, apart from CR^M337V^ (**P = 0.018 HCs, *P = 0.049 TDP-43^M337V^, *P = 0.038 TDP-43^I383T^; two-way ANOVA with Sidak’s multiple comparison test, *F* = 12.12). (**D**) A significant decrease in the percentage of stationary mitochondria is detected in transduced TDP-43^M337V^ (*P = 0.041) and TDP-43^I383T^ (***P = 0.0005, *F* = 16.08). (**E**) Percentage of iPS-MNs with mitochondria in dynamic pause is significantly increased by DCTN1 expression in TDP-43^I383T^ (****P < 0.0001) (two-way ANOVA with Sidak’s multiple comparison test, *F* = 11.49). Data are presented as violin plots (*n* = 2 independent differentiations for all iPS-MNs with recordings from at least 3 independent microgrooves).

In order to analyse whether increasing protein expression of dynactin-1 is sufficient to normalize axonal transport in TDP-43 iPS-MNs, we transduced the iPS-MNs with a DCTN1-GFP lentiviral construct on Day 25 and measured axonal transport on Day 33. Expression of DCTN1-GFP was similar among the HCs, TDP-43 iPS-MNs and CR^M337V^ isogenic control (Fig. 6A, Supplementary Fig. 8C). Analysis of axonal transport showed DCTN1-GFP doubled the percentage of retrograde running mitochondria in TDP-43^M337V^ (1.09% compared to 0.53%) and TDP-43^I383T^ (1.22% compared to 0.53%) in the presence of glutamate (two-way ANOVA, *P* < 0.05) and improved the speed of retrograde transport compared to HCs (Fig. 6B and C). The expression of DCTN1-GFP significantly reduced the percentage of stationary mitochondria in TDP-43^M337V^ by 3.4% and in TDP-43^I383T^ by 5.9% (two-way ANOVA, *P* < 0.05), thereby improving overall axonal transport dynamics (Fig. 6D and E).

Similar to untransduced iPS-MNs, no differences were detected in axonal transport at baseline conditions, indicating exposure to glutamate is necessary to reveal functional disturbances. In our model system, overexpression of DCTN-1 increased the overall movement of mitochondria and improved the speed of retrograde transport.

## Discussion

In this study, we demonstrate that TDP-43 mutations reduce mitochondrial bioenergetics, such as ATP production and respiration, which is partly mediated by an interaction with the beta subunit of complex V, ATP synthase and COX5A. Cytochrome c oxidase (COX) is the terminal enzyme of the mitochondrial respiratory chain, and it catalyzes the transfer of electrons from reduced cytochrome c to molecular oxygen. It is believed to play an important role in the regulation of age-related oxidative phosphorylation,^42^ and early studies have demonstrated a decrease in COX activity in motor neurons from patients with sporadic ALS.^43^

Furthermore, we show that TDP-43 mutations in iPS-MNs from ALS patients contribute to the reduction in retrograde axonal transport of mitochondria and endosomes, most likely through a combinatorial effect of reduced bioenergetics and reduction of DCTN-1/dynein. Dynactin subunit 1 is a large subunit of the dynactin complex, which regulates the activity of the molecular motor complex dynein by binding the two complexes together on the microtubules.^44^ A reduction in both mRNA and protein levels of DCTN-1 has been reported in sporadic ALS patients, and mutations in this gene are associated with disease, indicating that altered expression of DCTN-1 may be involved in the pathophysiological process.^16,27,45,46^

Previous studies have shown that TDP-43 mutations contribute to mitochondrial changes seen in neurodegeneration. Our group has recently reported reduced mitochondrial Ca^2+^ buffering capacity in both TDP-43^M337V^ iPS-MNs and C9ORF72 iPS-MNs from ALS patients.^30,31^ Fibroblasts from TDP-43^A382T^ ALS patients were also reported to have a fragmented mitochondrial network and decreased membrane potential,^5^ while C9-ALS fibroblasts were reported to have mitochondrial morphological modifications, such as mixed populations of elongated, short mitochondria and functional modifications, such as increased ATP production and respiration, and membrane hyperpolarization.^5^ These studies are consistent with our findings of smaller mitochondria in iPS-MNs from patients with TDP-43 mutations.

While we detect a transcriptional upregulation of ATPB and COX5A in TDP-43 patient iPS-MNs and TDP-43^M337V^ mouse ES-derived MNs, the functional output in mitochondrial bioenergetics is reduced in human cells. Our findings are consistent with a recent study that showed abnormalities in the electron chain machinery in human iPS-derived MNs from C9ORF72 patients, where low basal respiration and maximal mitochondrial respiration were detected.^37^ At the same time, we observe increased co-localization of mutant TDP-43^M337V^ with ATPB and COX5A in human iPS-MNs, indicating a potential compensatory mechanism where the interaction of TDP-43 reduces the efficiency of ATP synthase and COX5A and induces transcriptional upregulation in a feedback mechanism. While we did not detect ATPB or COX5A in the immunoprecipitation of endogenous TDP-43 in our iPS-MNs, we cannot exclude a direct interaction. The complexes pulled down by IP are mainly cytosolic complexes, which are readily accessible for precipitation, while ATPB and COX5A are located in the inner mitochondrial membrane, which may not be easily pulled down in such an experiment and may account for the discrepancy between our co-localization imaging experiment and the immunoprecipitation. Furthermore, a modest increase in binding propensity of mutant forms of TDP-43 to ATPB or COX5A may be masked by the heterozygous presence of normal TDP-43 in these iPS-MNs from patients. Further investigations using *in silico* experiments would be worthwhile to explore the possibility of direct binding.

TDP-43 has also previously been found to bind to the mitochondrial outer membrane, suggesting that it may physically interfere with mitochondria and potentially impair its transport.^8,47^ Mitochondrial transport in neurons is tightly coordinated by microtubule-based transport mediated mainly by the motor protein complex kinesins in anterograde transport and dynactin/dynein complexes in retrograde transport.^48,49^ Here, we show that retrograde axonal transport is disrupted in iPS-MNs carrying the M337V and I383T mutations in TDP-43, and this is mediated by a reduction in the largest dynactin subunit, DCTN-1 or p150^Glued^. A mutation in the p150 subunit of dynactin was previously linked to ALS, and reductions in its expression levels were reported in the spinal cords of sporadic ALS patients.^46^ DCTN-1 binds directly to dynein and microtubules, allowing dynein to travel long distances along the microtubules. We also found that dynein is downregulated in TDP-43^M337V^, contributing to the reduction in retrograde speed and further impairing mitochondrial and endosomal retrograde transport. In our model system, there is no difference in the percentages of anterograde and retrograde travelling mitochondria in patients compared to the controls, but only in the speed of transport, which indicates that cargo binding to the microtubule system occurs successfully, but the stability of the complexes may be affected leading to a reduction in speed. Since the transport is also ATP dependent, the reduction in ATP production, coupled with low levels of dynein/DCTN-1 in patient MNs, could account for the reduced speed observed in the retrograde direction. Despite the overall reduction in ATP production, anterograde axonal transport remains unaffected, indicating that reduction in DCTN-1 plays an essential contributory role to the specificity of axonal speed reduction in the retrograde direction. In line with this observation, we demonstrate here that overexpression of DCTN-1 in mutant iPS-MNs partially rescues axonal transport deficits, improving both speed of retrograde axonal transport and percentage of moving mitochondria. Recently, mutant DCTN-1 was shown to directly bind to the C-terminal domain of TDP-43 and act as a regulator of its aggregation propensity.^50^ In another report, the interplay between DCTN-1 and TDP-43 pathology was highlighted, showing that DCTN-1 knockdown increases TDP-43 cytoplasmic aggregation and accelerates the formation of ubiquitin-positive cytoplasmic inclusions, acting as a modifier of stress granule dynamics.^51^ Taken together, these studies and our current results indicate there may be a feedback loop where different dynamics of interaction between TDP-43 and DCTN-1 exacerbates disease phenotypes and pathology.

In line with our results, the earliest disease-related event observed in a TDP-43^A315T^ mouse was a reduction in retrograde mitochondrial axonal transport, which later led to accumulation of mitochondria in axon terminals and fragmentation.^7^ The primary function of retrograde transport in neurons is the removal of damaged organelles from the distal axons and, presumably, to target them for degradation in the soma,^52^ indicating that mitophagy may be impaired as a result of reduced retrograde transport. Furthermore, disrupting retrograde mitochondrial transport in zebrafish was shown to lead to impairments in presynaptic motor axon function, demonstrating that retrograde transport plays a key role in the homeostatic distribution of mitochondria.^53^

We also report here a downregulation of KIF5A and KIF5B in patient iPS-MNs with TDP-43 mutations, despite no differences in the speed or percentages of anterograde moving mitochondria. Mutations in KIF5A were identified in ALS patients in a large GWAS study,^54^ and truncation of the C-terminus in KIF5A led to a disruption in mitochondrial localization in the axons in a zebrafish model,^55^ highlighting the importance of cytoskeletal defects in the pathogenesis of ALS. We speculate that the absence of overt impairments in anterograde transport is due to compensatory mechanisms. The kinesin superfamily consists of 15 classes of kinesins, with over 45 known kinesins expressed in mammalian cells.^40^ The family of Kinesin-1 is the main anterograde transporter of mitochondria,^56^ and we have identified in our RNA sequencing, and consequently confirmed by immunoblotting, downregulation of two members of this family (KIF5A and KIF5B). While still under debate, kinesin-3 members KIF1B and KLP6 have also been shown to transport mitochondria anterogradely *in vitro*.^57^ As we do not detect transcriptional changes in these KIFs and KIF5C shows normal levels of expression, it indicates that they may compensate for the downregulation of KIF5A and KIF5B and contribute to maintaining the normal speed of anterograde mitochondrial transport. Furthermore, efficient dynein activity was shown to require kinesin-1, which is needed to deliver dynein from the soma to the synapses.^58^ The downregulation of both kinesin-1 and dynein may have an additive effect on retrograde transport, explaining why anterograde transport of mitochondria may be less affected in our neurons. Anterograde mitochondrial transport may also provoke a better compensatory response of motor proteins within the timeframe of our short recordings (2 min), and we cannot exclude the possibility that sustained exposure to glutamate and neuronal activity will eventually reduce the speed of anterograde transport as well. Furthermore, we do not detect differential expression of adaptor proteins for anterograde transport (TRAK6, Miro1/2), indicating that KIFs may bind more efficiently to mitochondria cargo compared to dyneins, which require dynactin-1.

Interestingly, correcting the M337V mutation by CRISPR/Cas9 significantly improved expression of DCTN-1 and reduced co-localization of ATPB:TDP-43, but it did not significantly improve the ATP production, mitochondrial basal respiration or expression of other motor proteins. We observe a general improvement in most of the phenotypes, or a partial rescue, where the isogenic control is not significantly different to the HCs, indicating that some improvement has occurred. This apparent lack of complete rescue could be attributed to persistent epigenetic modifications, which may continue to be present despite genetic correction, or potentially other secondary mutations (SNPs). There are also additional pathways that may play a role and that are not directly related to the mutations, such as modifiers of disease. While generating isogenic controls that share a genetic background to the patient iPSC lines is a significant asset to determine mutation-driven disease phenotypes, the interpretation is more complicated in oligogenic diseases, such as ALS. To circumvent some of these issues, creating additional isogenic iPSC lines from other patients with the M337V mutation will be necessary to identify which phenotypes are attributable solely to the mutation. However, it is very likely that the absence of complete phenotype rescue is due to the complex nature of the disease and the regulatory systems associated with it.

## Conclusion

Our study shows a novel association of mutant TDP-43 with reduction in the transport motor proteins DCTN1 and dynein, resulting in a significant downregulation of retrograde axonal transport. A reduction in ATP production further contributes to the impairment in axonal transport, which is coupled with an interaction between TDP-43 and the mitochondrial subunit beta of ATP synthase and COX5A with significant downstream effects on mitochondrial bioenergetics. The correction of the M337V mutation by CRISPR/Cas9 does not significantly improve these phenotypes, indicating that further disease modifiers may be involved in the observed deficiencies. We report here that lentiviral overexpression of DCTN-1 is sufficient to improve retrograde axonal transport dynamics.

## Supplementary Material

fcae350_Supplementary_Data

## Data Availability

Full RNA sequencing data are available at NCBI GEO accession number GSE139144. Interactome data for mouse ES-MNs are available at PRIDE accession number PXD010354.
