# Supplementary material for: Dynactin-1 mediates rescue of impaired axonal transport due to reduced mitochondrial bioenergetics in amyotrophic lateral sclerosis motor neurons
Source: Brain Commun. 2024 Oct 5;6(5):fcae350. doi: 10.1093/braincomms/fcae350 (PMC11495216; doi:10.1093/braincomms/fcae350)
Supplement: fcae350_Supplementary_Data [file fcae350_supplementary_data.pdf]

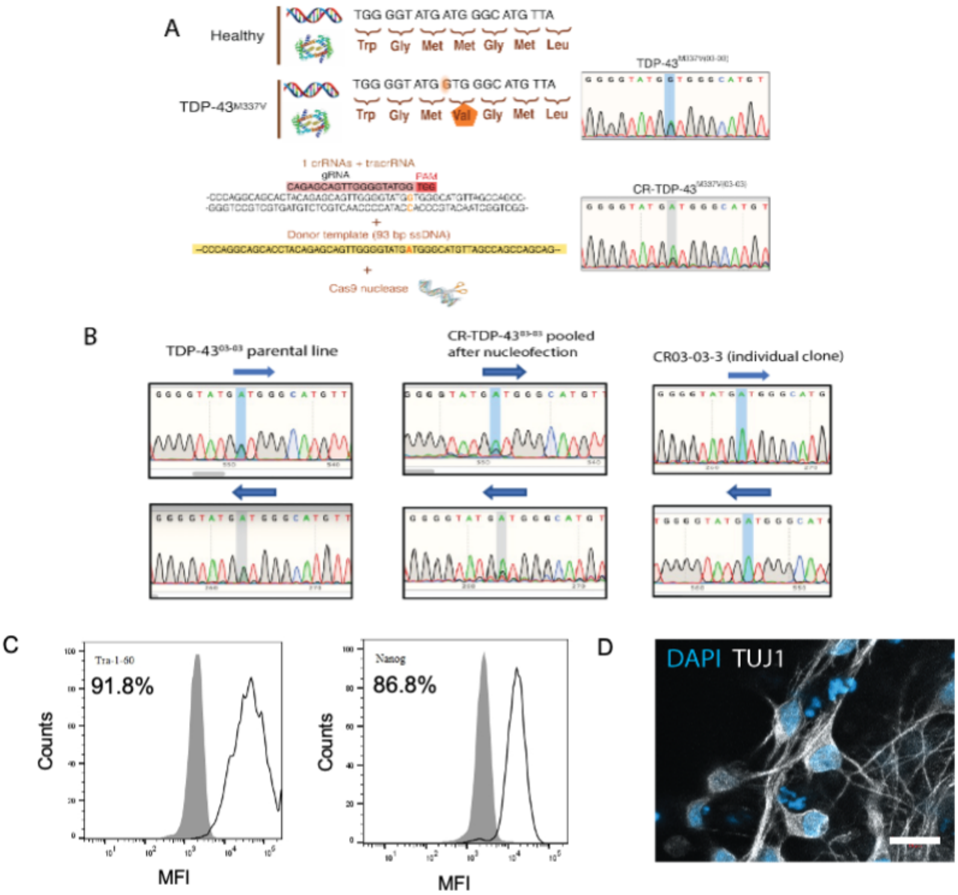

Supplementary Figure 1: Quality control of CRISPR/Cas9 isogenic line generated for TDP-43^{M337V} (line TDP-03-03). A) Design of the strategy for correcting M337V mutation by CRISPR/Cas9. B) Sequencing results of the parental line, pooled cells after transfection and an inidividually picked clone, showing successful correction of the mutation. C) Single Nucleotide Polymorphism (SNP) chip array shows normal karyotype, without any rearrangements. C) CR^{M337V} has normal pluripotency markers by FACS (MFI = median fluorescence intensity). D) Immunostaining showing successful differentiation of the isogenic line, CR^{M337V}, to neurons (Tuj1). Scale bar = 20 µm.

A

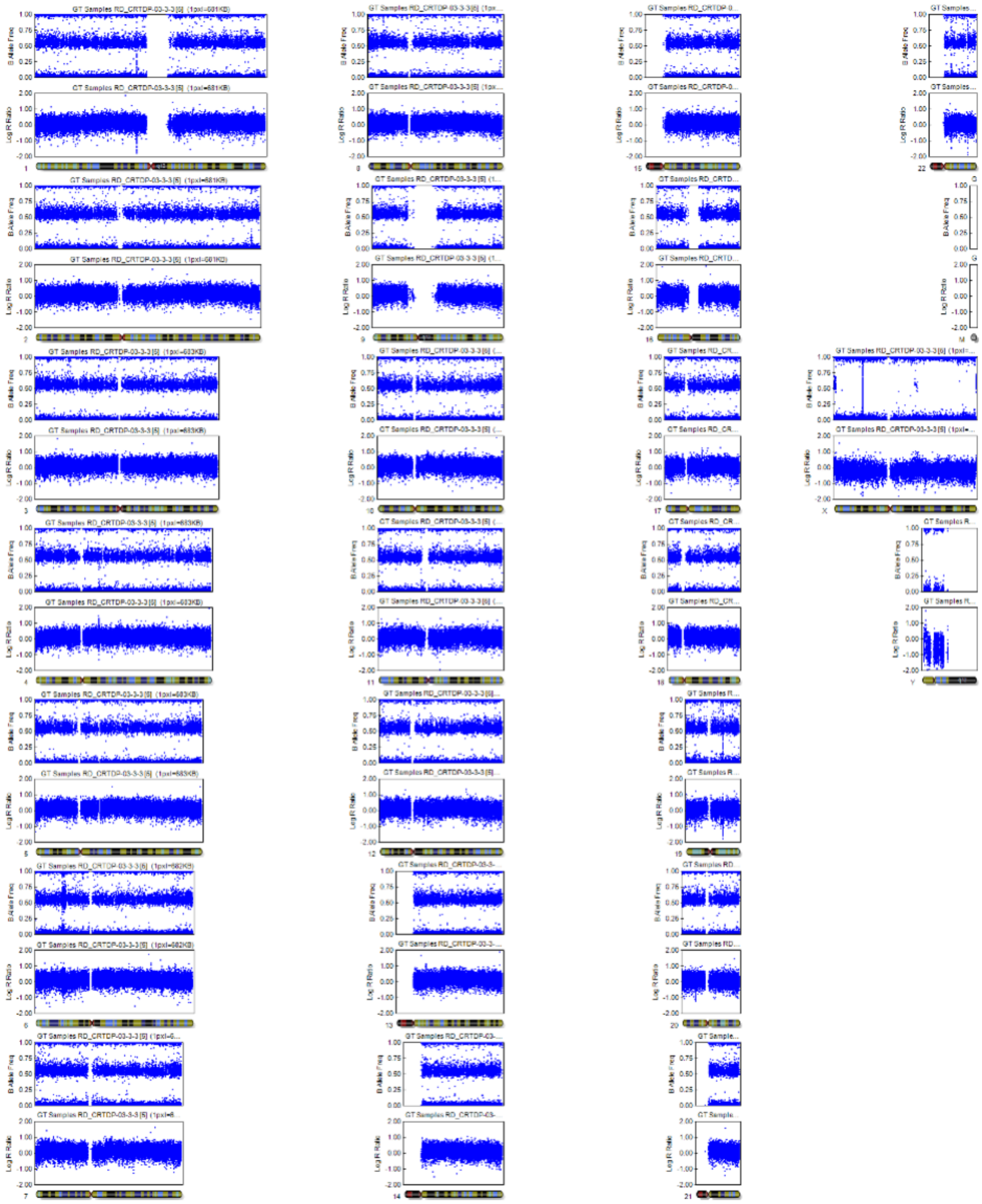

Supplementary Figure 2: Single Nucleotide Polymorphism (SNP) chip array shows normal karyotype, without any rearrangements.

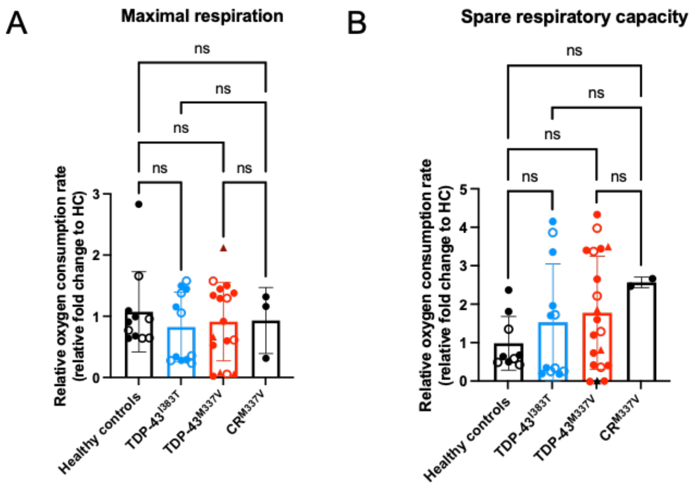

Supplementary Figure 3: Maximal respiration and spare respiratory capacity are not affected by TDP-43 mutations in iPS-MNs. No differences are detected by Seahorse XFe in A) maximal respiration or B) spare respiratory capacity between TDP-43 patients and healthy controls (n=4 independent differentiations for healthy controls and TDP-43^{M337V} iPS lines; n=3 for TDP-43^{I383T}; n=1 for CR^{M337V}; data points represent technical replicates (2-3) for each iPS line from independent differentiations; symbols represent different iPS lines). Statistical tests performed on both graphs: One-Way ANOVA with Dunnett's multiple comparisons test.

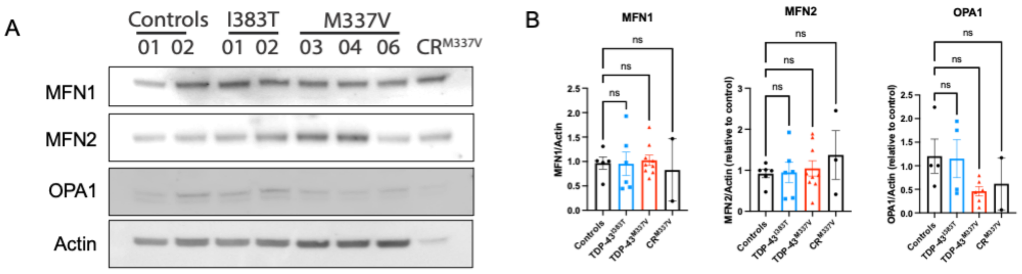

Supplementary Figure 4 : Mitochondrial fusion is not altered in patient iPS-MNs. A) Immunoblotting for MFN1, MFN2 and OPA1 show B) no differences in expression between healthy controls, TDP-43 iPS-MNs or isogenic line. Data points represent individual iPS lines from 2-3 independent differentiations (n.s., One-Way ANOVA with Dunnett's post hoc test for multiple comparisons). See Supplementary Figure 15 for uncropped blots.

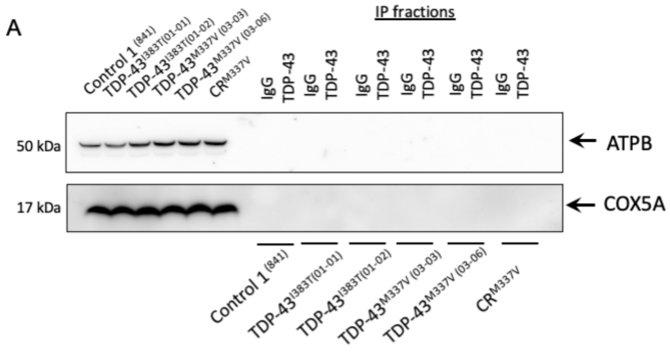

Supplementary Figure 5: Immunoprecipitation of TDP-43 in iPS-MNs. A) Immunoprecipitation using TDP-43 shows that ATPB and COX5A are not detected in the IP (n=2 independent differentiation, with 2 iPS-lines for I383T and 2 iPS lines for M337V). See Supplementary Figure 12 for uncropped blots.

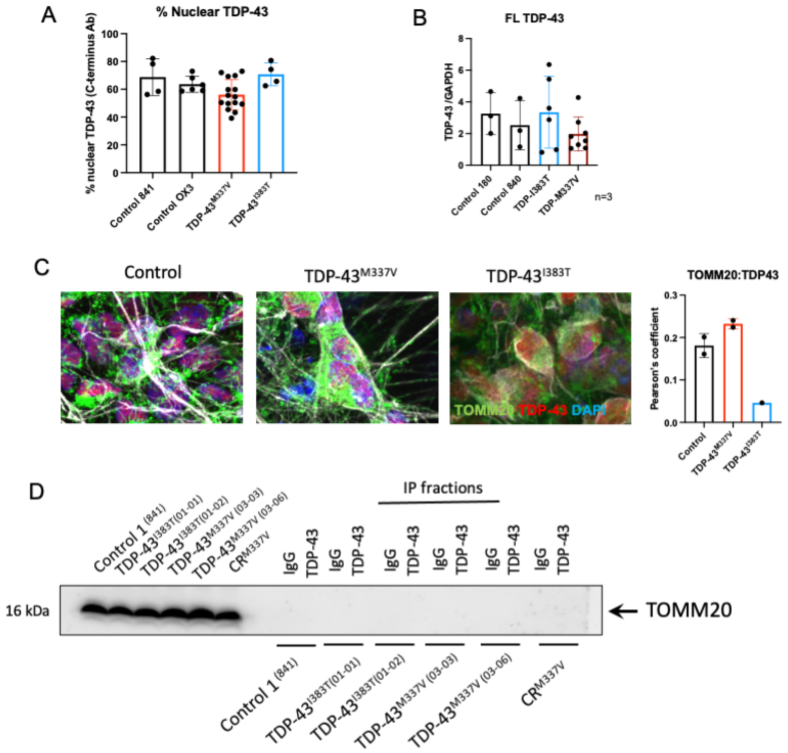

Supplementary Figure 6: TDP-43 is not mislocalized to the cytoplasm in patient MNs. A,B) Percentage of nuclear TDP-43 is not significantly different between patient MNs and healthy controls in analysis of immunostaining (n=2 independent differentiations, One-Way ANOVA with Dunnett's *post-hoc;* data points represent individual iPS lines). C) TDP-43 does not significantly co-localize with TOMM20 in the mitochondria at baseline (n.s., One-Way ANOVA with Dunnett's test for multiple comparisons; n=2 independent differentiations, data points represent values for each iPS-line per differentiation) and D) TOMM20 is not co-immunoprecipitated by TDP-43. See Supplementary Figure 12 for uncropped blots.

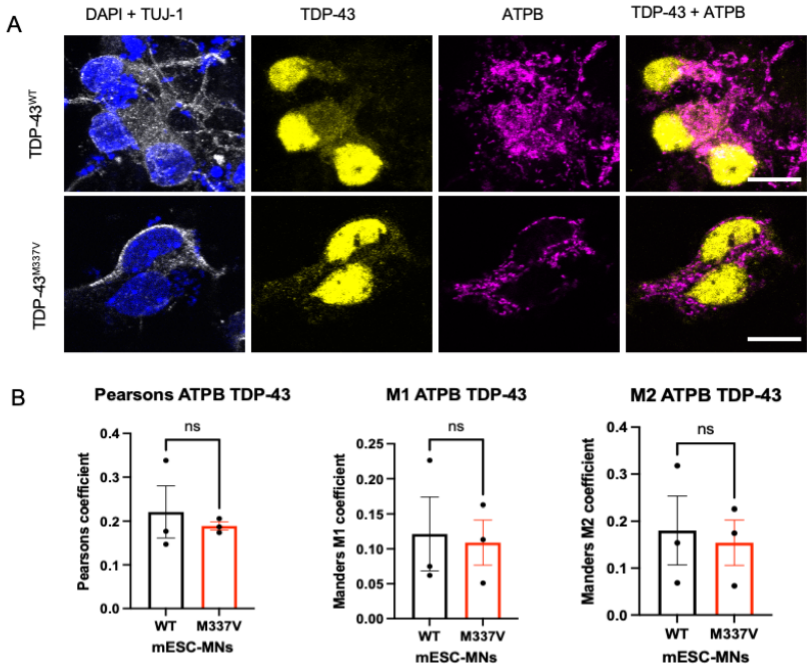

Supplementary Figure 7: No co-localization is detected in mouse ES-MNs from TDP-43^{M337V}-Ypet between TDP-43 and ATPB. A) Immunostaining for ATPB in mouse ES-MNs shows no significantly co-localization between TDP-43 and ATPB (B). Pearson's and Manders coefficients for co-localization (n.s. Student's *t* test). Scale bar = 10 µm.

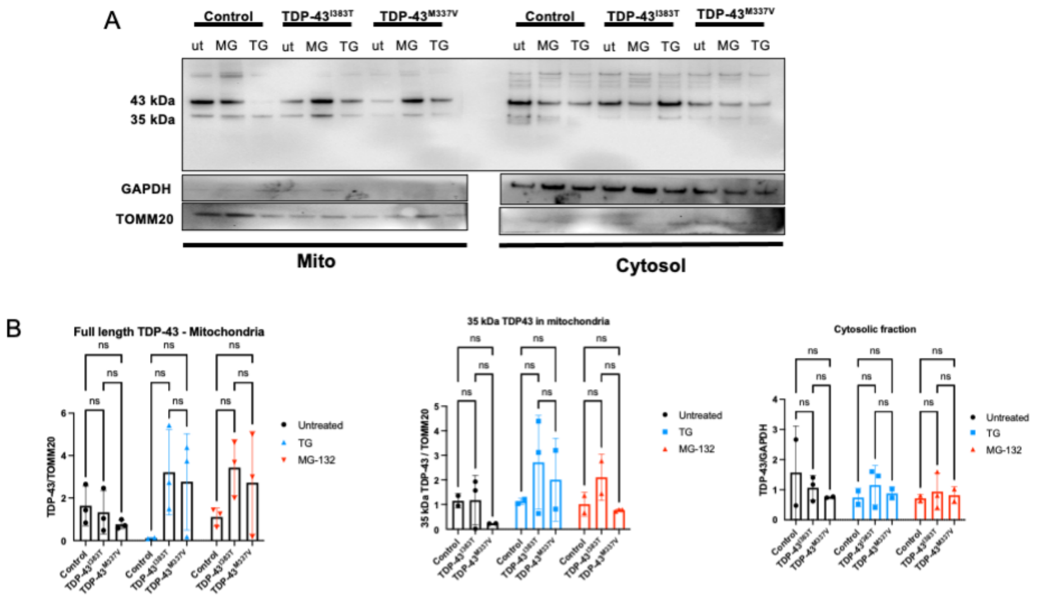

Supplementary Figure 8: C-terminal fragments of TDP-43 are not overexpressed in the mitochondria of mutant TDP-43 iPS-MNs. A,B) Immunoblotting for TDP-43 (C-terminus) for mitochondrial and cytosolic fractions shows no differences in the expression levels of full-length TDP-43 and 35 kDa fragment of TDP-43 in mitochondria or cytosol of patient MNs compared to healthy controls (n.s., Two-Way ANOVA with Dunnett's multiple comparisons test, n=2 independent differentiations). Data points represent individual iPSC lines, with 2 iPS lines for TDP-43^{M337V}, 2 iPS lines for TDP-43^{I383T} and 2 healthy control iPS lines. See Supplementary Figure 13 for uncropped blots.

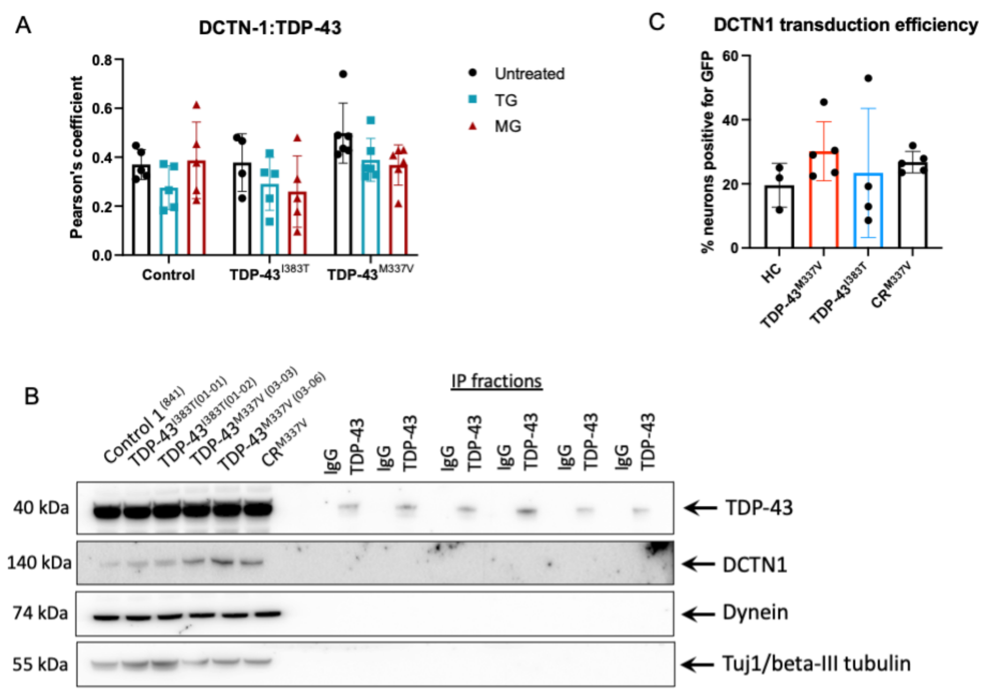

Supplementary Figure 9: DCTN-1 co-localization with TDP-43 and DCTN1 transduction efficiency. A) DCTN1 does not co-localize with TDP-43 under stress conditions by immunostaining (n.s., Two-Way ANOVA). B) Co-immunoprecipitations show that TDP-43 does not pull down DCTN1 and dynein. C) DCTN1-GFP transduction efficiencies are similar among the genotypes (n.s., One-Way ANOVA). Data points represent technical replicates of individual lines, with at least 1 replicate per differentiation. See Supplementary Figure 14 for uncropped blots.

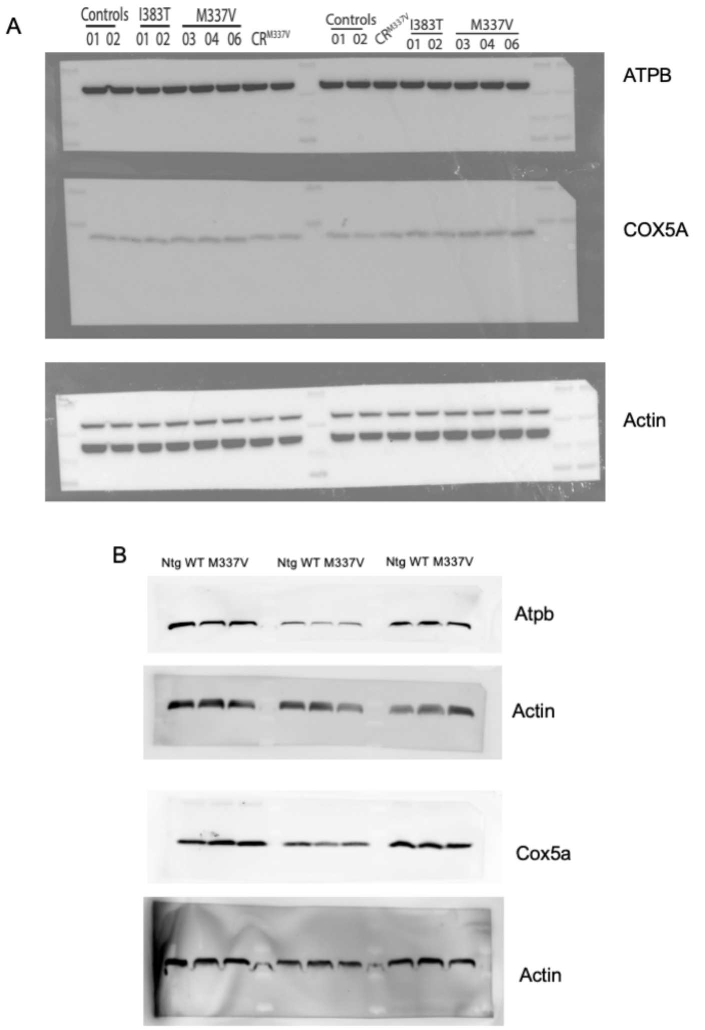

Supplementary Figure 10: Uncropped western blots for ATPB and COX5A. A) Full size western blots for ATPB, COX5A and actin in human iPS-MNs (related to Fig 2 C,D); B) Full size western blots for AtpB, Cox5a and their corresponding actin blots in mouse ES-MNs (related to Fig 2 G,H). Bands for WT and M337V from the first differentiation (left samples) were used in Fig 2G (Atpb and corresponding actin) and bands for WT and M337V from the middle samples were used in Fig 2H (Cox5a and corresponding actin).

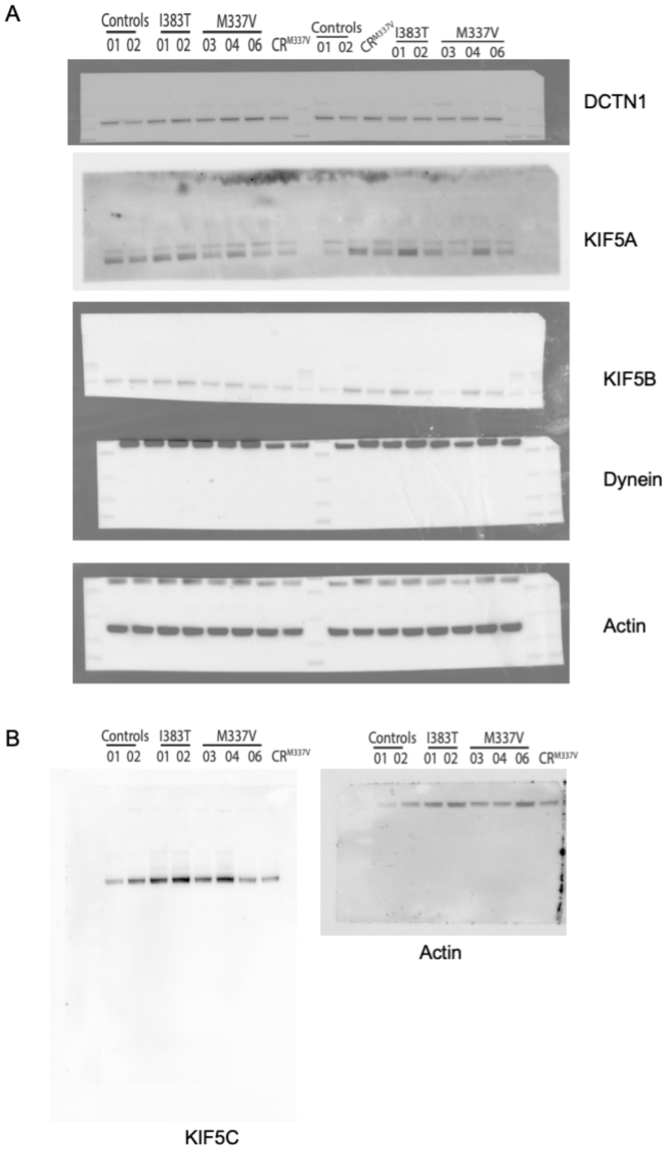

Supplementary Figure 11: Uncropped western blots for motor proteins. A) Uncropped western blots for DCTN1, Dynein, KIF5A, KIF5B and actin (related to Figures 5 B, D, F) and B) for KIF5C and its corresponding actin (related to Figure 5F).

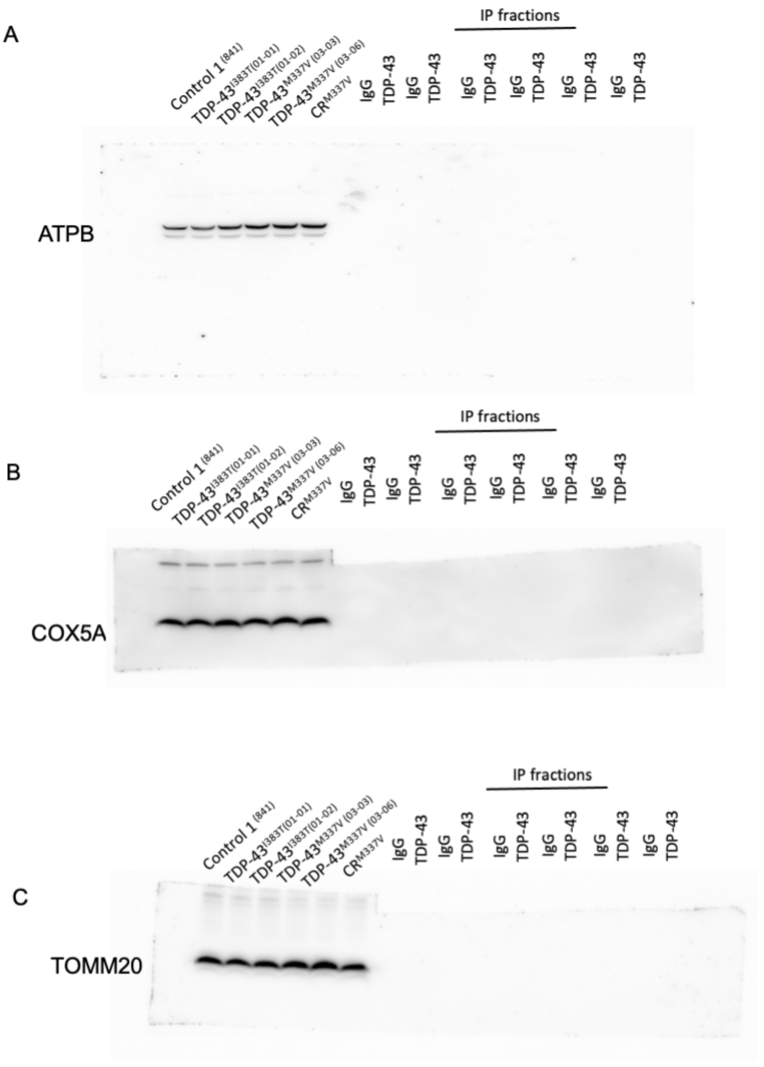

Supplementary Figure 12: Uncropped western blots. A) Full size western blots related to Supplementary Figure 5. B) Full size western blots related to Supplementary Figure 6D.

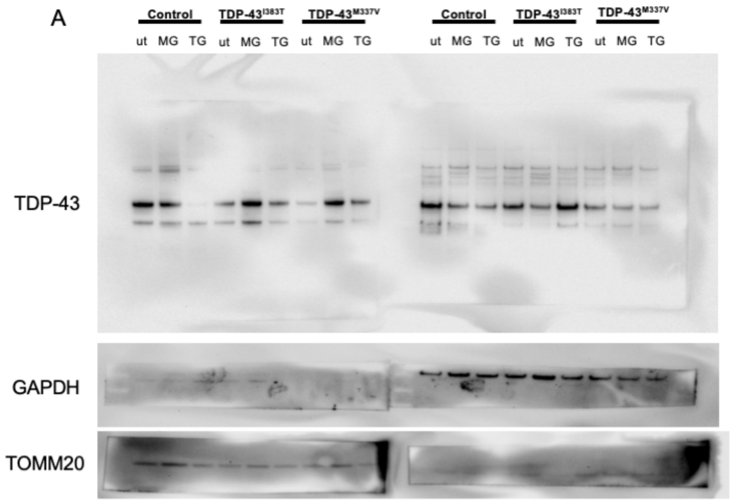

Supplementary Figure 13: Uncropped western blots. A) Full size western blots related to supplementary figure 8.

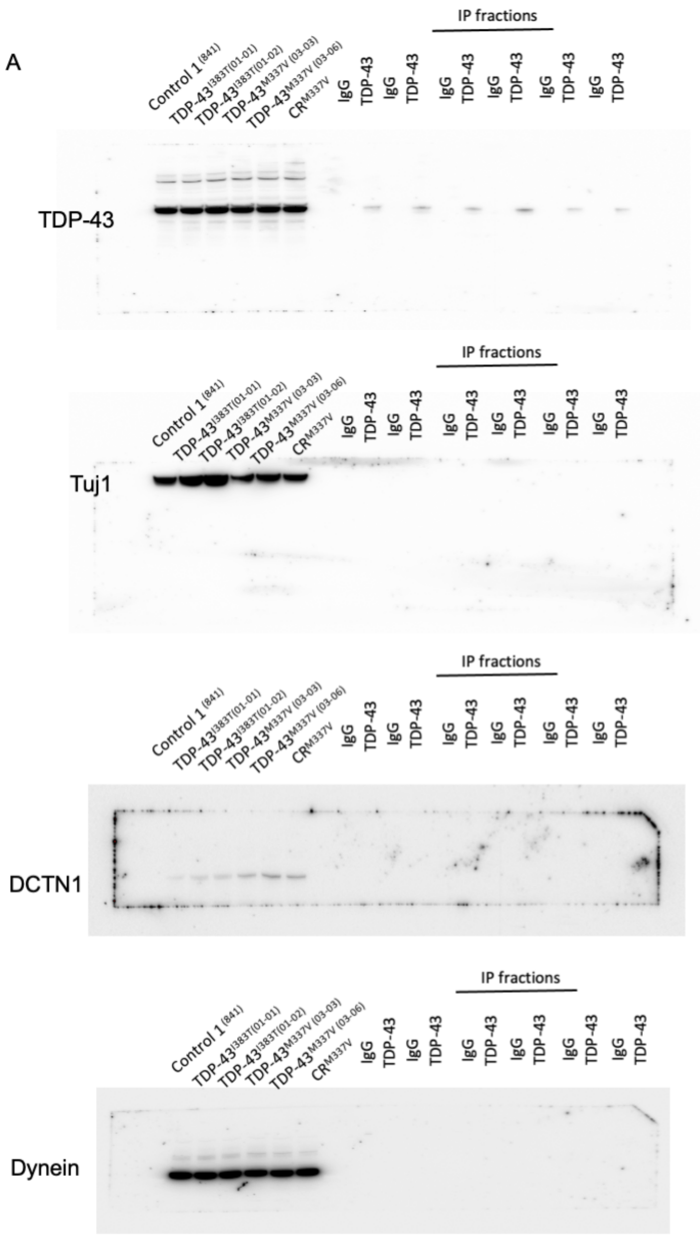

Supplementary Figure 14: Uncropped western blots. A) Full size western blots related to Supplementary Figure 9.

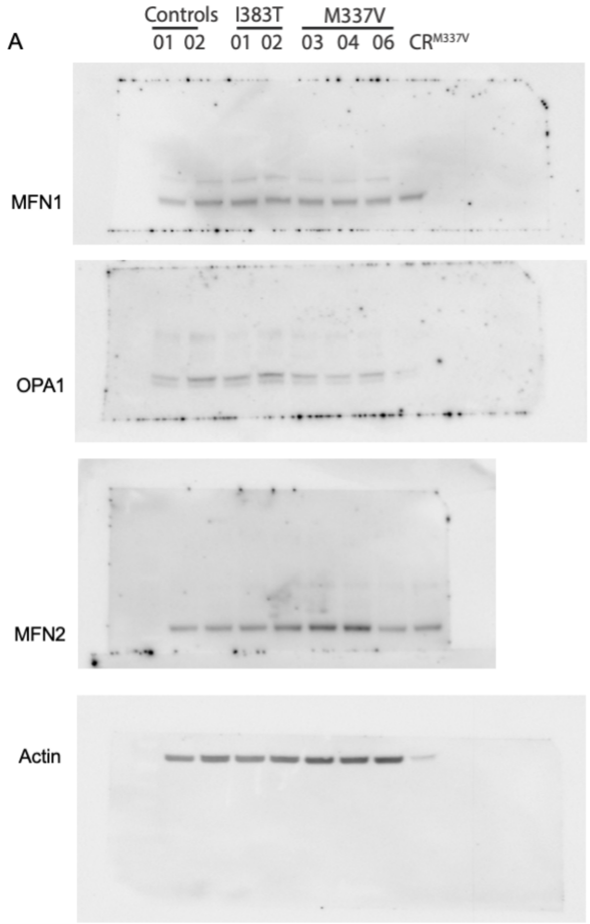

Supplementary Figure 15: Uncropped western blots. A) Full size western blots related to Supplementary Figure 4.

Supplementary Table 1: Induced pluripotent stem cell lines used in the study

Patient/control	iPS lines	Mutation	Age	Gender
SFC 841 (control 1)	03-01	-	36	Male
OX3 (control 2)	08	-	49	Male
TDP-03	03, 04 and 06	M337V in TARDBP	57	Male
TDP-01	01-01 and 01-02	I383T in TARDBP	60	Male
CR-03	-03	CRISPR/Cas9 isogenic controls of TDP-03-03		

Supplementary Table 2: Sequences of primers and guide RNAs used in the study

Name of oligo	Sequence (5'-3')
Forward check – exon 6	CAGGTGGCTTTGGGAATCAG
Reverse check – exon 6	ACTCCACACTGAACAAACCA
Alt-R crRNA(gRNA)	GCUCUGUAGUGCUGCCUGGGGUUUUAGAGCUAUGCU
Donor template ssDNA	CTGACTGGTTCTGCTGGCTGGCTAACATGCCCATCATAC CCCAACTGCTCTGTAGTGCTGCCTGGGCGGCAGCCATC ATGGCTGGATTAATGC
